# Artificially sporulated *Escherichia coli* cells as a robust cell factory for interfacial biocatalysis

**DOI:** 10.1038/s41467-022-30915-2

**Published:** 2022-06-06

**Authors:** Zhiyong Sun, René Hübner, Jian Li, Changzhu Wu

**Affiliations:** 1 Department of Physics, Chemistry and Pharmacy, University of Southern Denmark, Campusvej 55, 5230 Odense, Denmark; 2 Institute of Ion Beam Physics and Materials Research, Ion Beam Center, Helmholtz-Zentrum Dresden - Rossendorf, Bautzner Landstrasse 400, 01328 Dresden, Germany; 3 School of Physical Science and Technology, ShanghaiTech University, 201210 Shanghai, China; 4 Danish Institute for Advanced Study (DIAS), University of Southern Denmark, Campusvej 55, 5230 Odense, Denmark

**Keywords:** Biocatalysis, Biosynthesis, Biotechnology

## Abstract

The natural bacterial spores have inspired the development of artificial spores, through coating cells with protective materials, for durable whole-cell catalysis. Despite attractiveness, artificial spores developed to date are generally limited to a few microorganisms with their natural endogenous enzymes, and they have never been explored as a generic platform for widespread synthesis. Here, we report a general approach to designing artificial spores based on *Escherichia coli* cells with recombinant enzymes. The artificial spores are simply prepared by coating cells with polydopamine, which can withstand UV radiation, heating and organic solvents. Additionally, the protective coating enables living cells to stabilize aqueous-organic emulsions for efficient interfacial biocatalysis ranging from single reactions to multienzyme cascades. Furthermore, the interfacial system can be easily expanded to chemoenzymatic synthesis by combining artificial spores with metal catalysts. Therefore, this artificial-spore-based platform technology is envisioned to lay the foundation for next-generation cell factory engineering.

## Abstract (web summary)

Whole-cell biocatalysis is of interest for the efficient production of a range of products but cell stability can be an issue. Here, the authors turn recombinant enzyme-producing *E. coli* into artificial spores to add protection from UV radiation, heating, and organic solvents by coating the cells with polydopamine to create robust cell factories.

## Introduction

In biology, cells have evolved defensive mechanisms against the unfavorable environment^1^. An inspiring example is the bacterial spores that are enveloped with multilayered barriers/coats to shield environmental stresses, e.g., UV radiation and chemical assault, for preserving DNA machinery^2–4^. Today, these defensive spores can be repurposed for synthetically useful biotransformation. For instance, endospores from *Bacillus subtilis* and *Saccharomyces cerevisiae* are engineered for catalysis in the presence of adverse radicals or organic solvents^5,6^. However, natural spores for catalysis are inherently limited by few available microbes and their metabolic dormancy with depressed enzyme activities^7,8^.

These limitations have spurred the current interest in artificial spores, in which vegetative cells are chemically coated by cytoprotective materials to endow durable cell-in-shell capsules, resulting in single-cell nanoencapsulation^9,10^ or individual surface-engineered microorganisms^11^. Distinct from naturally occurring dormant spores, artificial spores are the encapsulated living cells that are not only robust but also metabolically active, thus holding great potential for biosynthesis^12–14^. In a pioneering study, Tang’s group prepared the silica coating on cyanobacteria to shield UV light stress for photosynthesis^15^. Since then, various microbes with their natural endogenous enzymes have been artificially sporulated for value-added chemicals production from asymmetric synthesis to renewable energy^16–18^. These successes verify the advantage of artificial spores for robust catalysis under challenging conditions, thus appealing for industrial operation. However, these previous efforts are mainly devoted to individual biosynthesis^17,18^, and artificial spores have never been developed into a generic synthetic platform. In this context, it is surprising that *Escherichia coli* (*E. coli*) cells, a model cell factory, have been rarely sporulated for synthetic purposes to date despite their capacity of high-level expression of heterologous enzymes with flexible metabolism pathways^19^. The only reported example is from the Ansorge-Schumacher group, who coated lyophilized *E. coli* cells with silicone for selective carboligation, which, however, caused significant cell death^20^. On the other hand, cell viability is essential to whole-cell catalysis, offering long-term enzyme activity and expensive cofactors regeneration^21^. Therefore, the development of viable *E. coli* cells-based artificial spores is desirable because they provide not only a robust microbial host but also a versatile cell factory, where various recombinant enzymes can be expressed for valuable chemical transformations.

**Fig. 1:**
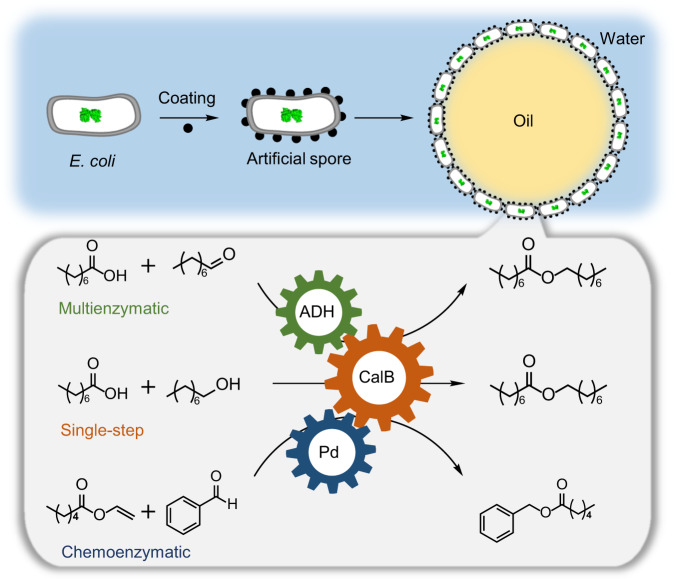
Schematic of the coating on *Escherichia coli* (*E. coli*) cells as artificial spores for interfacial biocatalysis. ADH alcohol dehydrogenase; CalB *Candida antarctica* lipase B; Pd palladium.

Here, we propose a simple yet general approach to preparing living *E. coli* cells based artificial spores via a biocompatible polydopamine coating and showcase their capacity for challenging interfacial catalysis (Fig. 1). In chemistry, many reactions are designed deliberately with catalysts at the interface of water-organic emulsions, which belongs to the process called “interfacial catalysis”^22^. Interfacial catalysis is an important research field because it offers a large surface area for fast reactions. However, its application is generally limited to stable catalysts due to the adverse interfacial environment, e.g., interfacial tension and shear force, which are deleterious to vulnerable catalysts, especially to biocatalysts^23–26^. Therefore, performing interfacial reactions is notoriously challenging in catalyst design, and there is no report of living *E. coli* cells for interfacial catalysis as a result of susceptible cellular structure and difficulty in maintaining intracellular enzyme activity. We opt to apply artificial spores for interfacial catalysis because its challenging environment is an ideal tool to evaluate the robustness of artificial spores. Furthermore, interfacial reactions have remarkable catalytic efficiency suitable for both polar and nonpolar substrates^27,28^, thus enabling to promote artificial spores for a broad-spectrum synthesis.

In this work, dopamine (DA) is chosen to coat the *E. coli* surface because of its proven biocompatibility and protectivity against external stresses. Another component, *N*-oleoyl dopamine (OLDA), is co-coated on cells to tailor their surface hydrophobicity. At the optimal ratio between DA and OLDA, the artificially sporulated cells can emulsify an organic-aqueous biphasic reaction medium into emulsions, creating a large interface area for reactions (Fig. 1). Challenged by this emulsion condition, the sporulation protects cells from environmental stresses while allowing recombinant enzymes in artificial spores for various biotransformation ranging from single-step reactions to multienzyme cascades. Furthermore, a chemo-enzymatic cascade by artificial spores and palladium particles is demonstrated. As a result, a robust versatile cell factory is enabled by the living *E. coli* cells based acritical spores, suggesting its fascinating perspective for future synthetic chemistry.

## Results and discussion

### Characterization of artificial spores

**Fig. 2:**
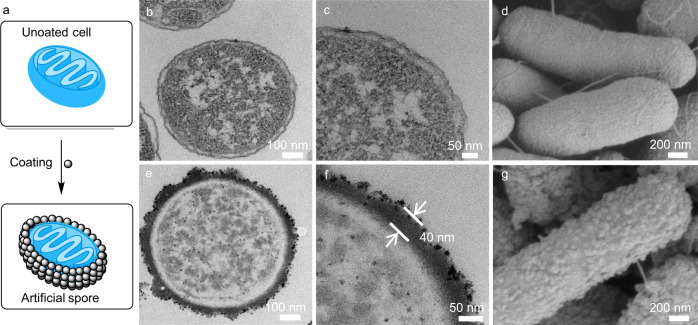
Characterization of artificial spores. **a** Scheme of the artificial sporulation on an *E. coli* cell. Cross-sectional bright-field transmission electron microscopy (TEM) images of (**b**, **c**) a native *E. coli* cell at different magnifications, as well as (**e**, **f**) an artificial spore at different magnifications. Scanning electron microscopy (SEM) images of (**d**) native *E. coli* cells and (**g**) artificial spores.

The sporulation process could be identified visually by the cell suspension turning into deep black color upon polydopamine coating^29^. After a washing step, UV-Vis and zeta potential analysis were conducted to characterize the physical property change of the cell surface. Strong absorption at 280 nm was observed on the artificial spores, which was in contrast to the control of uncoated cells with no specific absorption (Supplementary Fig. 1). Furthermore, the coating resulted in the zeta potential changing from negative to positive due to the significant presence of amino groups on the coated cells (Supplementary Fig. 2). To gain more insights into the shell, cross-sectional transmission electron microscopy (TEM) was employed to investigate the structures of artificial spores and uncoated cells (Fig. 2a). A black shell was observed on the artificial spore (Fig. 2e, f) with an average thickness of 40 nm (Fig. 2e), while there was no such shell found on uncoated cells (Fig. 2b, c). This structure difference indicates the successful formation of a polydopamine shell on *E. coli* cells. Further investigation by scanning electron microscopy (SEM) was performed to study the morphology changes of the coated cell surface. As shown in Fig. 2d, uncoated cells have comparatively smooth membranes, while on the artificial spores, cells were closely packed by polydopamine nanoparticles (Fig. 2g). Consequently, these microscopic studies strongly suggest the occurrence of artificial sporulation, which provides the base for further investigation of artificial spores’ functions.

### Biocompatibility of the sporulation process

**Fig. 3:**
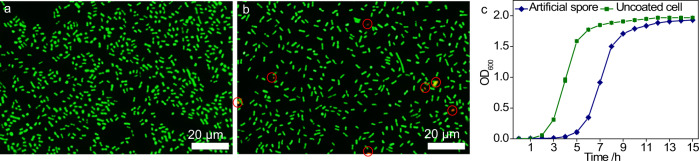
Evaluation of the biocompatibility of the sporulation process. Live/dead assay for (**a**) native *E. coli* cells and (**b**) artificial spores; green - live cells; red - dead cells. **c** Growth curve of native *E. coli* cells (green curve) and artificial spores (blue curve). The results in (**c**) are the average values of three parallel experiments. The error bars represent the standard deviations of three parallel measurements, *n* = 3.

After physical/microscopic studies, the sporulation was evaluated for its biocompatibility using live/dead assays^30^. The uncoated *E. coli* cells were taken as the control, whereby only green fluorescence (live cells) was observed (Fig. 3a). For the artificial spores, the majority of cells were in the green color (>97%) with only a few in red (dead cells) (Fig. 3b). This indicates that our coating is biocompatible with minimized cell destruction, which is in line with previous observations on other cells^29^. This positive finding motivated us to study the proliferative ability of artificial spores (Fig. 3c). Although the artificial spores showed a delayed growth curve compared to the uncoated *E. coli* cells, the two kinds of cells had similar growth ability, which is consistent with previous reports^31^. The above results collectively demonstrate that the sporulation is a mild process, allowing for cell division even after the coating.

### Protectability from external stresses

**Fig. 4:**
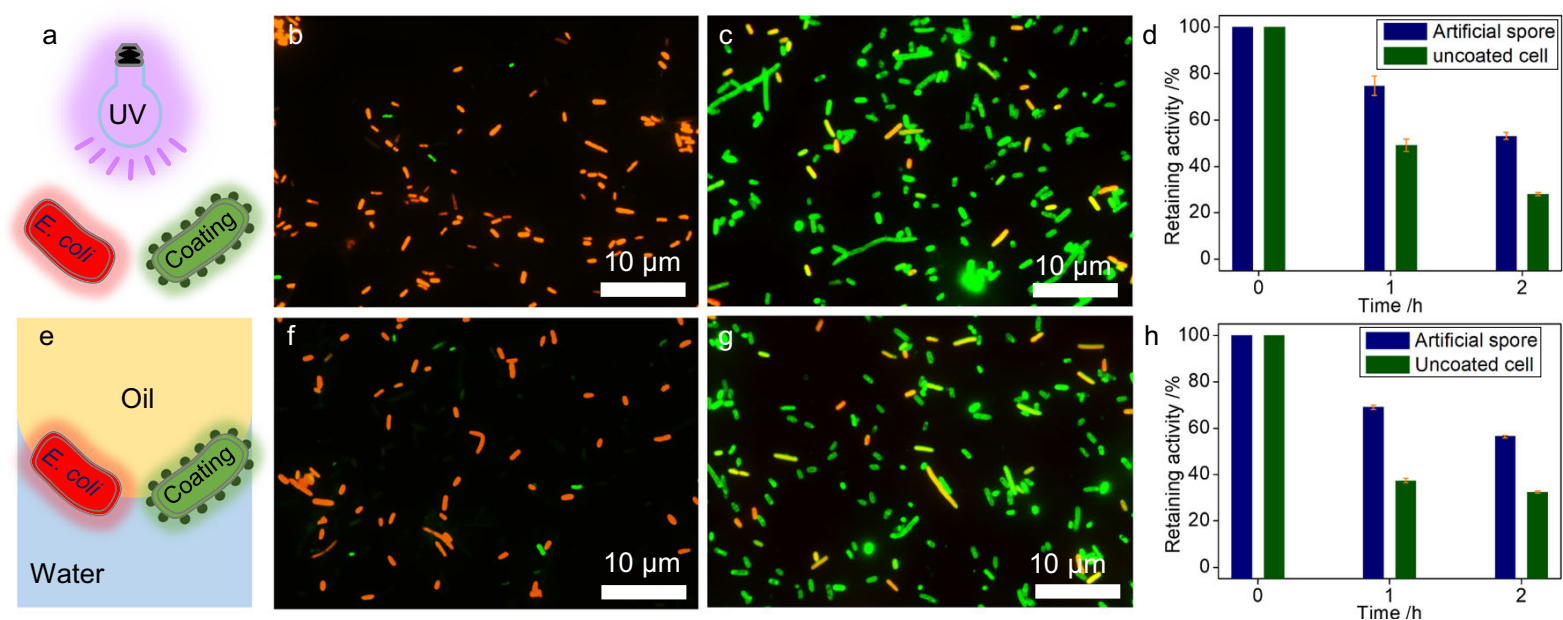
The protectability of artificial spores from external stresses. **a** Scheme of uncoated *E. coli* cell and artificial spore under ultraviolet (UV) light irradiation. Live/dead assay of (**b**) uncoated cells and (**c**) artificial spores after 2 h UV treatment; green - live cells; red - dead cells. **d** Benzaldehyde lyase (BAL) stability under UV treatment; blue columns - artificial spores; green columns - uncoated cells. **e** Scheme of uncoated cell and artificial spore under interfacial stress (i.e., toluene-water biphasic condition). Live/dead assay of (**f**) uncoated cells and (**g**) artificial spores treated by 2 h interfacial stress; green - live cells; red - dead cells. **h** BAL stability under interfacial stress; blue columns - artificial spores; green columns - uncoated cells. The results in (**d**, **h**) are the average values of three parallel experiments. The error bars represent the standard deviations of three parallel measurements, *n* = 3.

The successful coating on *E. coli* cells encouraged us to explore their protectability from environmental stresses. In the first experiment, both coated and uncoated cells were exposed to UV radiation (Fig. 4a), which is a known antibacterial condition to *E. coli* cells^32^. The live/dead assay showed that 2 h UV treatment caused significant death to the uncoated cells but only slightly affected to the coated cells (> 85% viability), suggesting the coating to be UV-protective (Fig. 4b, c). Subsequently, both cells were subjected to a water-toluene biphasic medium to evaluate if the coating could withstand the interfacial stress (Fig. 4e), which is important to their future use in interfacial catalysis. Interestingly, the interfacial exposure led to 90% cell death for uncoated cells (Fig. 4f), but the sporulated shell largely protected cells from the interfacial environment with over 80% cell viability (Fig. 4g). These results illustrate the robustness of the artificial spores, which would be of great interest for their catalytic assessment. In this context, we overexpressed benzaldehyde lyase (BAL) in *E. coli* cells, followed by the polydopamine coating, and then treated them under four types of harsh environmental conditions. In all cases, enzymes in artificial spores showed better stability than in the native cells. For example, artificial spores maintained almost two-fold enzyme activity than native cells under 2 h UV exposure (Fig. 4d). A similar result was observed when treating cells with a water-toluene medium, whereby enzyme activity of artificial spore was also much higher (Fig. 4h). Moreover, the sporulated shell protected cells from the heating and acetonitrile solvent, resulting in their higher enzyme activity compared to the control samples (Supplementary Figs. 3, 4). The above findings, therefore, proved the protectability of the artificially sporulated cells against diverse environmental stresses, suggesting their potential as durable catalysts for challenging reaction conditions.

### Emulsion formation by artificial spores

**Fig. 5:**
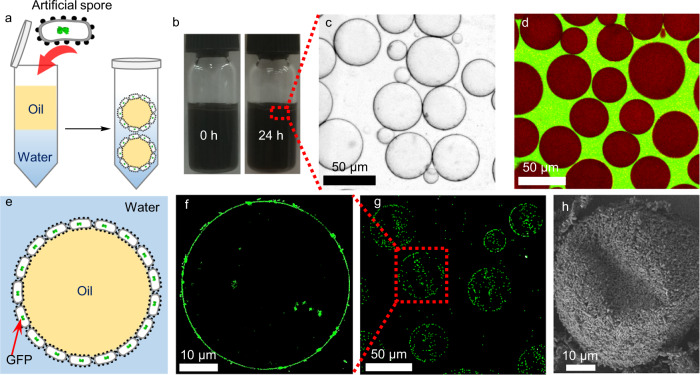
Characterization of Pickering emulsion. **a** Scheme of emulsion preparation. **b** Emulsion appearance at 0 h and 24 h. **c** Optical microscopy image of the emulsions. **d** Confocal laser scanning microscopy (CLSM) image of the oil-in-water emulsions; red – organic phase, green – aqueous phase. **e** Scheme of emulsion droplet stabilized by artificial spores with green fluorescent protein (GFP). CLSM images of (**f**) an individual emulsion droplet and (**g**) an emulsion stabilized by artificial spores containing GFP. **h** SEM image of an individual emulsion droplet surface.

To explore interfacial biocatalysis, a prerequisite is to coat the cells with the appropriate surface amphiphilicity for emulsion formation. As such, the sporulation was tailored for the optimal cell surface by adjusting the coating condition using concentrations of OLDA from 0.4 to 0.65, 1.05, and 1.4 mg/mL, resulting in different ratios of OLDA to DA. Since OLDA and DA had different polarities, their co-coating in different ratios resulted in several hydrophobized cell surfaces (Supplementary Fig. 5). Subsequently, emulsions were prepared by mixing these coated cells with a biphasic mixture of cyclopentyl methyl ether (CPME) and PBS buffer, assisted with hand-shanking (Fig. 5a). The emulsion stability test suggested the most stable emulsions (in 24 h) obtained at 0.65 mg/mL OLDA (Fig. 5b, Supplementary Figs. 6, 7), which was thus used in the following investigations. The resultant emulsions were characterized for their size and type using microscopical approaches. A typical emulsion with 10–70 µm diameters was directly observed by visible-light microscopy (Fig. 5c), while the emulsion type was determined by identifying the location of two distinct dyes, Nile red (red, organic-solvent-soluble) and fluorescein isothiocyanate-labelled hyperbranched polyglycerol (green, water-soluble), in the emulsions. Confocal laser scanning microscopy (CLSM) images showed that an oil-in-water emulsion was typically formed (Fig. 5d). To get further insight into the cell distribution within the emulsions, we prepared the emulsions using artificial spores overexpressed with green fluorescent protein (GFP) (Fig. 5e). The CLSM images (Fig. 5f, g) showed that the fluorescent cells were mainly allocated on the emulsion surface, demonstrating that the emulsions were indeed stabilized by artificial spores. A similar observation was made by SEM analysis (Fig. 5h, Supplementary Fig. 8), where artificial spores fully assembled on the outer layer of the emulsion surface with micron-sized pores. This closely packed porous structure illustrates the sufficient amount of cells on the emulsions with pores facilitating structures for substrate access, which should be beneficial to a fast mass transfer in catalysis.

### Proof-of-concept of interfacial biocatalysis

**Fig. 6:**
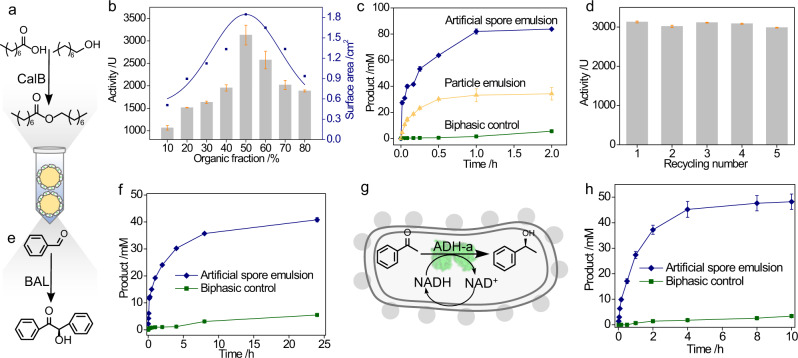
Investigation of interfacial biocatalysis for single-step reactions. **a** A model esterification reaction catalyzed by CalB. **b** Enzyme activity (left axis) and surface area (right axis, blue curve) of emulsions with different oil-to-water ratios. **c** Reaction profiles of CalB from artificial spore emulsion (blue), silica particle emulsion (yellow) and unemulsified biphasic system (green). **d** Reuse of artificial spores. **e** A benzoin condensation reaction by BAL. **f** BAL-catalyzed reaction profiles in artificial spores emulsion (blue) and biphasic control (green). **g** A cofactor regeneration within artificial spores for the reduction of acetophenone by alcohol dehydrogenase *Rhodococcus ruber* (ADH-a), NADH: nicotinamide adenine dinucleotide hydrogen (reduced), NAD^+^: nicotinamide adenine dinucleotide (oxidized). **h** ADH-a-catalyzed reaction profiles in artificial spores emulsion (blue) and biphasic control (green). The results in (**b**–**d**, **f**, **h**) are the average values of three parallel experiments. The error bars represent the standard deviations of three parallel measurements, *n* = 3.

Given the unique composition and structure of the emulsions, they were of high interest for their use in catalysis. As a proof-of-concept, we started with a robust enzyme, *Candida antarctica* lipase B (CalB) that was overexpressed in *E. coli* cells prior to emulsion formation. A model esterification reaction was established for catalysis evaluation, as shown in Fig. 6a. Since interfacial catalysis is known for its dependency on emulsion preparation^33^, we optimized the emulsions in terms of their oil-to-water ratio and found that this ratio had a significant impact on the enzyme activity (Fig. 6b). Increasing the organic phase fraction from 10 to 50% dramatically enhanced the enzyme activity, but its further increase led to decreased activity. This activity variation was explained by the formation of different emulsions that could be observed from the CLSM images in Supplementary Fig. 10. The interface area of these different emulsions can be calculated from their droplet sizes and numbers^34,35^. Typically, a large interface area resulted in higher enzyme activity, and the best activity was achieved with 50% organic fraction (Fig. 6b). Moreover, we investigated the influence of different concentrations and hydrophobicity of artificial spores on their interfacial catalytic performance. Typically, increasing artificial spores from 3.2 to 6.4 × 10^9^ cells/mL led to the faster initial reaction rate, but their further increase did not improve the bioconversion over 2 h (Supplementary Fig. 11). Besides, cells surface properties were optimized for the best bioconversion with OLDA 0.65 mg/mL modification (Supplementary Fig. 12). Therefore, an optimal Pickering emulsion could be prepared from 6.4 × 10^9^ cells/mL with 0.65 mg/mL OLDA coating. Next, this optimal emulsion was compared with two benchmark controls: i) a biphasic reaction medium with uncoated cells, and ii) a classic emulsion stabilized by silica nanoparticles (NPs) and with uncoated cells. For a fair comparison, all reactions were performed under the same conditions with the same amount of *E. coli* cells. Figure 6c showed that artificial spores had a much better catalytic performance than both controls. Specifically, CalB activity in artificial spores was 300-fold higher than in biphasic medium and 3 times greater than in particle-stabilized emulsions (Supplementary Fig. 13). This activity enhancement is ascribed to the large interface area of the emulsions as well as the protective effects of the artificial spores. Since the artificial spores are stable and do not lose their integrity during centrifugation, they could be recycled five times without significant loss of enzyme activity (Fig. 6d). Such reusability was also confirmed by the fact that recycled cells from the emulsions were still metabolite-active (Supplementary Fig. 14). Therefore, this proof-of-principle study illustrates the highly reactive and recyclable artificial spores for interfacial catalysis.

As *E. coli* is the most popular platform for recombinant protein expression, we were interested in if other types of enzymes could be also suitable for our artificial spores. To this end, we overexpressed two other different enzymes in *E. coli* for interfacial reactions. The first one was benzaldehyde lyase (BAL), which was a relatively sensitive enzyme and was used for C-C coupling reaction (Fig. 6e). Experimental data showed that in the artificial spores, BAL was still active under the interfacial environment, achieving 200-time higher activity than the biphasic medium with uncoated cells (Fig. 6f, Supplementary Fig. 15). With this success, we then studied a cofactor-dependent enzyme, alcohol dehydrogenase *Rhodococcus ruber* (ADH-a), in coated *E. coli* cells (Fig. 6g). Interestingly, ADH-a was not only active for 10 h but also performed without the supplement of nicotinamide adenine dinucleotide (NADH) in emulsions (Fig. 6h), illustrating that the cofactor was regenerated from the metabolite process of living cells. This cofactor regeneration eventually enabled artificial spores to achieve better catalysis than the control (Supplementary Fig. 16). Taken together, we have shown that artificially sporulated *E. coli* cells could act as a synthetic cell factory for single-step interfacial reactions with diverse enzymes ranging from stable to sensitive and cofactor-dependent enzymes.

### Multienzyme cascades

**Fig. 7:**
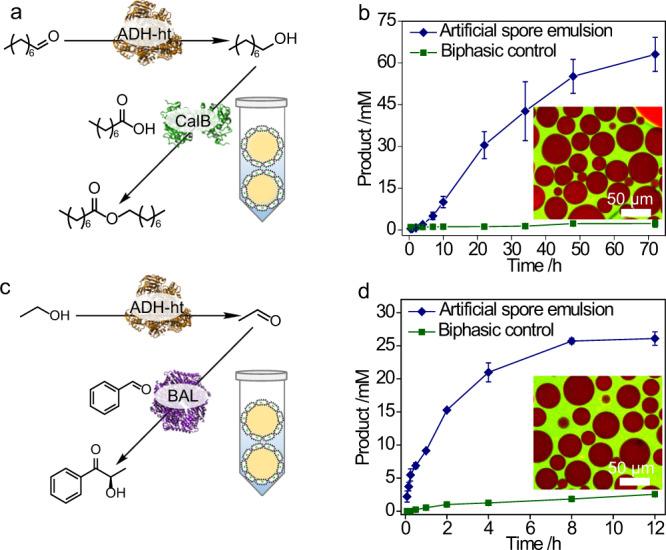
Investigation of multienzyme cascades. **a** Cascade reaction with alcohol dehydrogenase from *Bacillus stearothermophilus* (ADH-ht) and CalB, **b** their reaction profiles in artificial spores emulsion (blue) and biphasic control (green), inset is the emulsion picture by CLSM; red – organic phase, green – aqueous phase. **c** Cascade reaction with ADH-ht and BAL, and (**d**) their reaction profiles in artificial spores emulsion (blue) and biphasic control (green), inset is the emulsion picture by CLSM; red – organic phase, green – aqueous phase. The results in (**b**, **d**) are the average values of three parallel experiments. The error bars represent the standard deviations of three parallel measurements, *n* = 3.

The success in single-step reactions motivated us to further explore the feasibility of using artificial spores for multienzyme cascades. For easy preparation, three cascade routes were designed with some of the enzymes studied above. In the first cascade, alcohol dehydrogenase from *Bacillus stearothermophilus* (ADH-ht) and CalB were respectively overexpressed in two different *E. coli* cells, which were then artificially sporulated for the two-step interfacial reaction (Fig. 7a). As a result of the protectability, the coating allowed the whole-cell reaction to being performed without the supplementation of the external cofactor, achieving much faster reactivity than the biphasic control with uncoated cells (Fig. 7b, Supplementary Fig. 17). After the first success, we aimed at using the artificial spores for more value-added production, and therefore, a second cascade with ADH-ht and BAL was performed for the production of optically active α-hydroxy ketone (Fig. 7c), which is a convenient building block in many organic synthesis^36^. Figure 7d showed that a faster reaction was observed by ADH-ht and BAL compared to the biphasic system (Supplementary Fig. 18). Lastly, we could also combine BAL and CalB into our artificial spores for the fast two-step interfacial reactions (Supplementary Fig. 19). The success in these three types of multienzyme cascades suggests that living *E. coli* cells based artificial spores can be easily extended from single to multienzyme reactions without the compromise of their reactivity.

### Chemoenzymatic cascades

**Fig. 8:**
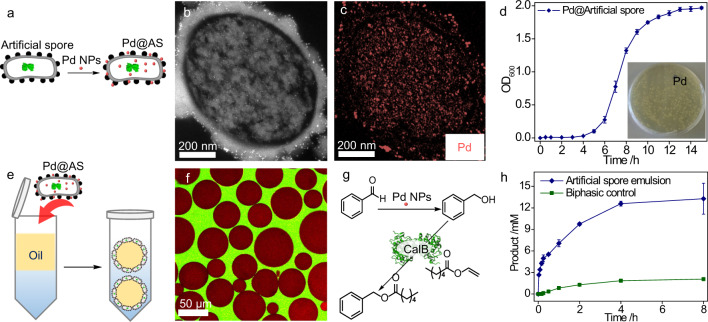
Investigation of chemoenzymatic cascades. **a** Schematic presentation of Pd-NPs-loaded artificial spores (Pd@AS); Pd-NPs: palladium nanoparticles. **b** High-angle annular dark-field STEM image of a Pd@AS cross-section; STEM: scanning transmission electron microscopy. **c** STEM-EDXS-based Pd (pink) element map; EDXS: energy-dispersive X-ray spectroscopy. **d** Growth curve of artificial spores, the inset showing their growth on agar plate. **e** Scheme of emulsion formation by Pd@AS. **f** CLSM image of an emulsion stabilized by Pd@AS; red – organic phase, green – aqueous phase. **g** Reaction scheme of chemoenzymatic cascades by Pd NPs and CalB, and (**h**) the reaction profiles in artificial spores emulsion (blue) and biphasic control (green). The results in (**d**, **h**) are the average values of three parallel experiments. The error bars represent the standard deviations of three parallel measurements, *n* = 3.

The combination of whole-cell and chemical catalysts is an attractive approach for developing multifunctional catalysts for advanced synthesis^37^. However, this combination remains a great challenge because of the mutual inactivation of both catalysts^38,39^. In particular, cells are prone to die in the presence of harmful chemical catalysts, accordingly leading to intramolecular enzyme inactivation^40^. To address this challenge, we utilized the protectability of artificial spores and constructed a hybrid catalyst system from them, in which palladium nanoparticles (Pd NPs) were in situ produced from artificial spores that contained CalB (Fig. 8a, see Methods for details). The successful loading of Pd NPs could be confirmed by scanning TEM imaging combined with energy-dispersive X-ray spectroscopy analysis (EDXS), as shown in Figure 8b, c. Interestingly, Pd NPs could be found both on the cell surface and inside the artificial spores. Their homogeneous distribution, however, raised our concern about the negative impact of intracellular Pd NPs to the cells. Hence, we assessed the cell proliferativity and growth ability after Pd NPs loading. Figure 8d showed that these Pd-treated cells had a similar growth profile and division behavior as normal artificial spores, indicating that the Pd NPs had only a minor effect on the cellular metabolite activity. Moreover, the presence of Pd NPs didn’t significantly change the cell surface morphology (Supplementary Fig. 20), but still allowed the artificial spores to stabilize an oil-in-water emulsion (Fig. 8e, f). The emulsion formation was then used for the chemoenzymatic reaction, as shown in Fig. 8g. The emulsion was able to catalyze a faster product formation with 350-fold higher activity than the biphasic control (Fig. 8h, Supplementary Fig. 21). This success indicates that artificial spores are a simple and robust platform that may be further developed by combining other chemical catalysts for different chemoenzymatic synthesis.

In summary, we developed a biocompatible polydopamine coating on *E. coli* cells, accordingly creating artificial spores that are featured with a cell-in-shell structure to protect the cells from various environmental stresses. This sporulation process is accomplished with only two chemical components, and thus can be easily adjusted to obtain an optimal cell surface for the formation of emulsions. On a broader perspective, this proof-of-principle study provides a facile method to the field of artificial spores where a plateau of other cells can be coated with an optimal surface for bio-related applications. Moreover, empowered by the protectability, these artificial spores are explored as a versatile cell factory, in which four different enzymes are overexpressed to catalyze interfacial reactions ranging from the single step to multienzyme cascades. In addition, we showcased that the catalytically active artificial spores are combined with Pd NPs for efficient chemoenzymatic cascades. With these successful demonstrations, we anticipate that our robust artificial spores can be further developed to address other challenges in synthetic cascades and biotransformation in general.

## Methods

### Materials

Commercial chemicals were used without further purification unless otherwise mentioned. Dopamine, oleic acid, and epoxy embedding medium kit were purchased from Sigma Aldrich. A bacterial viability kit was purchased from Thermo Fisher. Sodium tetrachloropalladate and sodium borohydride were purchased from abcr GmbH. The preparation of cell cultures and the expression of enzymes were described in the supplementary information.

### Synthesis of *N*-oleoyl dopamine (OLDA)

The OLDA was synthesized according to the procedure described in the literature^41^. Typically, oleoyl chloride was added dropwise to the solution of dopamine (1.2 eq) and triethylamine (4 eq) in dichloromethane, and the crude product was purified by silica gel column chromatography (hexane-ethyl acetate) to obtain the OLDA.

### Preparation of artificial spores

In general, *E. coli* cells were suspended in 20 mL Tris buffer (10 mM, pH 8.5) to a final optical density (OD_600_) of 2.0. Then the mixture (40 mg) of DA and OLDA with different ratios was dissolved in 2 mL dimethyl sulfoxide (DMSO) and added dropwise to coat the cells for 2 h. Subsequently, the coated cells were collected by centrifugation and washed 3 times. The cells were finally collected and re-suspended in 5 mL PBS buffer (10 mM, pH 7.4) for emulsion formation.

### Interfacial catalysis

A typical emulsion was prepared for interfacial catalysis by mixing 0.5 mL phosphate-buffered saline (PBS buffer, 10 mM, pH 7.4 and 1 mM glucose) containing artificial spores and organic phase of cyclopentyl methyl ether (CPME), followed by handshaking. The reactions were initiated by adding substrates to the emulsions with a final concentration of 100 mM. All samples were prepared in triplicate and analyzed by gas chromatography (GC). The data were averaged for plotting at each time interval, and standard deviations were calculated as the error bars.

### Pd NPs on artificial spores

An in situ reduction method was taken to produce Pd NPs in/on artificial spores. In a typical protocol, a sodium tetrachloropalladate solution was added to the artificial spore medium to reach a final concentration of 1 mM, and the mixture was then kept for 15 min. After three-time washing with PBS buffer (10 mM, pH 7.4), a sodium borohydride solution was subsequently provided with a final concentration of 1 mM for reducing the ion to Pd NPs for 15 min. Finally, the Pd-loaded artificial spores were washed 3 times with PBS buffer (10 mM, pH 7.4) and collected for further use.

### Statistics and reproducibility

For SEM and TEM in Fig. 2b–g, two independent measurements were conducted with similar results. For cell viability assays in Figs. 3a, b, 4b, c, f, g, Supplementary Figs. 3a, b, and 4a, b, two independent measurements were performed with similar results. For the emulsion characterizations in Figs. 5c, d, f–h, 8b, c, f, Supplementary Figs. 7, 8a, b, 10, 14a, and 20, two independent measurements were conducted with similar results.

### Reporting summary

Further information on research design is available in the Nature Research Reporting Summary linked to this article.

## Supplementary information

Supplementary Information

Reporting Summary

## Data Availability

Data generated and analyzed during this study are included in this article and its Supplementary Information. Source data is available for Figs. 5c, f, h, 6b, 6d, 7h and Supplementary Fig. 19c, e in the associated source data file. Source data are provided with this paper.

## Source data

Source Data
