# Supplementary material for: Artificially sporulated Escherichia coli cells as a robust cell factory for interfacial biocatalysis
Source: Nat Commun. 2022 Jun 6;13:3142. doi: 10.1038/s41467-022-30915-2 (PMC9170730; doi:10.1038/s41467-022-30915-2)
Supplement: Supplementary file 1 — Supplementary Information [file 41467_2022_30915_MOESM1_ESM.pdf]

Supplementary Information

Artificially Sporulated *Escherichia coli* Cells as a Robust Cell Factory for Interfacial Biocatalysis

Zhiyong Sun,[1] René Hübner,[2] Jian Li,[3] Changzhu Wu[1,4*]

[1] Department of Physics, Chemistry and Pharmacy, University of Southern Denmark, Campusvej 55, 5230 Odense, Denmark; Email: wu@sdu.dk

[2] Institute of Ion Beam Physics and Materials Research, Ion Beam Center, Helmholtz-Zentrum Dresden - Rossendorf, Bautzner Landstrasse 400, 01328 Dresden, Germany

[3] School of Physical Science and Technology, ShanghaiTech University, 201210 Shanghai, China

[4] Danish Institute for Advanced Study (DIAS), University of Southern Denmark, Campusvej 55, 5230 Odense, Denmark

Table of Contents

1. Instruments 3
2. Enzyme expression 4
2.1 Expression of *Candida antarctica* Lipase B 4
2.2. Expression of benzaldehyde lyase (BAL) 6
2.3. Expression of alcohol dehydrogenase from *Rhodococcus ruber* (ADH-a) 6
2.4. Expression of alcohol dehydrogenase from *Bacillus stearothermophilus* (ADH-ht) 6
3. Sample preparation for electron microscopy 7
4. Cell viability assays 8
5. Protecability against external stresses 8
5.1 UV 8
5.2 Interfacial stress 9
5.3 Acetonitrile 9
5.4 Heating 9
6. Catalytic performance 10
6.1. Emulsions with different oil-to-water ratios 10
6.2. Interfacial catalysis with CalB 11
6.3. Interfacial catalysis with different concentrations of artificial spores 11
6.4. Interfacial catalysis with different hydrophobicity of artificial spores 12
6.5. Interfacial catalysis with BAL 12
6.6. Interfacial catalysis with ADH-a 13
6.7. Multienzyme cascade with ADH-ht and CalB 13
6.8. Multienzyme cascade with ADH-ht and BAL 14
6.9. Multienzyme cascade with CalB and BAL 14
6.10. Chemoenzymatic cascade with palladium nanoparticles and CalB 14
7. Experimental data 15
8. References 27

1. Instruments

Static water contact angle measurements were carried out with the Drop Shape Analysis System DSA 10 from Krüss. Prior to the measurement, powder samples were pressed into flat disc according to the literature,[1] and subsequently transferred to the device for the measurement via a glass slide. A drop of water (5 µL) was placed on the flattened sample and the image was immediately captured in computer for analysis. For each sample, the measurement was performed in triplicate.

UV-visible spectra were recorded on a Cary 5000 UV-Vis-NIR spectrophotometer (Agilent Technologies) at room temperature using a 10-mm quartz cell.

Scanning electron microscopy (SEM) analysis was performed using a S-4800 microscope (Hitachi) operated at an accelerating voltage of 1 kV.

Transmission electron microscopy (TEM) was performed on a Jeol JEM 1400 Plus instrument equipped with a Jeol Ruby camera and the Picture Overlay Program for correlative light electron microscopy (CLEM).

Furthermore, bright-field TEM images were recorded on a Titan 80-300 (FEI) microscope operated at an accelerating voltage of 300 kV. High-angle annular dark-field scanning transmission electron microscopy (HAADF-STEM) imaging and spectrum imaging based on energy-dispersive X-ray spectroscopy (EDXS) were performed at 200 kV with a Talos F200X microscope equipped with an X-FEG electron source and a Super-X EDXS detector system (FEI). Prior to TEM analysis, the specimen mounted in a high-visibility low-background holder was placed for 2 s into a Model 1020 Plasma Cleaner (Fischione) to remove contamination.

Optical microscope and fluorescence microscope images were recorded on Olympus Provis AX70.

Gas Chromatography (GC) analysis was carried out on a Shimadzu GC-2010 Plus high-end gas chromatography machine (software version: Shimadzu GCsolution 2.31), equipped by AOC-20i/s auto injector/auto sampler and BPX5 column (length: 25 m, inner diameter: 0.22 mm). The data were organized with Microsoft Excel 2016, then plotted with Origin 8.

Zeta potential analysis was performed using a Zetasizer Nano-ZS (Malvern Instruments Inc.) at room temperature. The same amount of coated (OLDA: 0.65 mg/mL) and uncoated cells were taken and dispersed in Milli-Q water for the zeta potential measurement, respectively.

2. Enzyme expression

2.1 Expression of *Candida antarctica* Lipase B

Candida antarctica lipase B (CalB) was expressed under the following conditions:

- Strain: *E. coli* BL21(DE3) containing pET22b_CalB-His (after expression CalB is localized in periplasm);
- Preculture: A preculture was prepared in lysogeny broth (LB) medium with 100 µg/mL ampicillin, incubation at 37 °C overnight;
- Culture: A main culture in autoinduction medium (recipe below, according to a previous publication)[2] was prepared with 100 µg/mL ampicillin, and with incubation at 20 °C overnight.

Supplementary Table 1. Autoinduction medium for CalB

	**Final concentration**	**Stock solution**	**per Liter**
Tryptone	1%		10 g
Yeast extract	0.5%		5 g
Na_2HPO_4	25 mM	50xM	20 mL
KH_2PO_4	25 mM		
NH_4Cl	50 mM		
Na_2SO_4	5 mM		
Mg SO_4	2 mM	1 M	2 mL
Trace metals	0.2x	1000x	0.2 mL
Glycerol	0.5%	50x 5052	20 mL
Glucose	0.05%		
D-Lactose	0.2%		

Add sterilized stocks to autoclaved tryptone + yeast extract (combined volume - 42.2 ml to 958ml of media) before use.

Supplementary Table 2. 50x salt stock solution

50x M	Molecular weight	Stock concentration	g/800 mL
Na_2HPO_4x7 H20	268.07 g/mol	1.25 M	268.07
KH_2PO_4	136.09 g/mol	1.25 M	136.09
NH_4Cl	53.49 g/mol	2.5 M	106.98
Na_2SO_4	142.04 g/mol	0.25 M	28.408
$MgSO_4$x7 H20	246.48 g/mol	1 M	123.24

Supplementary Table 3. 50x 5052 stock solution

50 X 5052	Stock	/500 mL
Glycerol	25%	125 mL
Glucose	2.5%	12.5 g
D-Lactose	10%	50 g

Supplementary Table 4. 1000x stock solution of trace metals

Metals	Molecular weight	Stock concentration	g/50 mL stock	stock/100 mL
$FeCl_3$	162.21 g/mol	0.1 M	0.811	50 mL
$CaCl_2$(x2 $H_2$0)	147.02 g/mol	2 M	14.7	1 mL
$MnCl_2$ (x4 $H_2$0)	197.91 g/mol	2 M	19.79	0.5 mL
ZnSO4 (x7H_2O)	287.54 g/mol	2 M	28.75	0.5 mL
$CoCl_2$ (x6H_2O)	237.93 g/mol	2 M	23.79	0.1 mL
$CuCl_2$	134.44 g/mol	2 M	13.44	0.1 mL
$NiCl_2$	129.62 g/mol	1 M	6.48	0.2 mL

Na_2MoO_4	205.92 g/mol	1 M	10.295	0.2 mL
Na_2SeO_3	172.94 g/mol	2 M	17.29	0.1 mL
H_3BO_3	61.83 g/mol	0.2 M	0.62	1 mL

2.2. Expression of benzaldehyde lyase (BAL)

BAL in *E. coli* cells was expressed according to the literature.[3] Typically, BAL was produced with the recombinant strain *E. coli* SG13009 containing pBAL-His6 plasmid. *E. coli* cells were incubated in LB/ampicillin medium overnight at 37 °C as the preculture. The preculture was then transferred to the main culture and incubated at 37 °C until reaching OD_{600} 0.6, whereby a final concentration of 0.8 mM isopropyl β-D-1-thiogalactopyranoside (IPTG) was added to induce the expression. After that, further incubation was carried out for another 16 h. The cells were collected by centrifugation (4 °C, 4193 x g., 10 min).

2.3. Expression of alcohol dehydrogenase from *Rhodococcus ruber* (ADH-a)

E. coli cells with ADH-a was obtained according to a published protocol.[4] *E. coli* BL21 containing pET22b+-ADH-a plasmid was used to expressed ADH-a. In principle, cells were first incubated in LB/ampicillin medium overnight at 37 °C as the preculture. The preculture was then transferred to the main culture and incubated at 37 °C until reaching OD_{600} 1.0, whereby the final concentrations of 1 mM IPTG and 0.43 mM $ZnSO_4$ were added to induce the expression. After that, further incubation was carried out for another 8 h at 15 °C. The cells were collected by centrifugation (4 °C, 4193 x g, 10 min).

2.4. Expression of alcohol dehydrogenase from *Bacillus stearothermophilus* (ADH-ht)

ADH-ht in *E. coli* cells was expressed according to a previous publication.[5] In a typical procedure, *E. coli* BL21 containing pET21a+-ADH-ht plasmid was incubated overnight at 37 °C as the preculture. The preculture was then transferred to the main culture and incubated at 37 °C until reaching OD_{600} 0.7, whereby a final concentration of 1 mM isopropyl β-D-1-thiogalactopyranoside (IPTG) was added to induce the expression. The cultures were shaken overnight at 20 °C, 120 rpm. After that, the cells were harvested by centrifugation at 4 °C, 4193 x g,10 min.

3. Sample preparation for electron microscopy

The *E. coli* cells (OLDA: 0.65 mg/mL for the coating) were dispersed in phosphate buffer (100 mM, pH 7.4) containing 2% glutaraldehyde for primary fixation at 4 °C overnight, and then the cells were collected and washed 3 times with Mili-Q water. The cells were further treated with osmium tetroxide (1% in Mili-Q water) for post-fixation at 4 °C for 2 h, followed by washing with Mili-Q water 3 times. After these treatments, the cells were dehydrated by a series of ethanol-in-water solutions for 15 min, with the volumetric ratio changed from 30 to 50, 70, 75, 90, 95, 100, and 100%. After dehydration, the cells are collected by centrifugation and used in the next sample preparation for SEM and TEM analysis.

For SEM, the dehydrated cells were dried with the critical-point drying technique according to the literature[6] and then coated by platinum for better conductivity.

For TEM, the dehydrated cells were first dispersed in three different mixture solutions containing propene oxide and embedding medium, in which the embedding medium content (v/v) was stepwise increased from 2:1 to 2:1 and 1:2, and cells were respectively incubated in each mixture for 1 h. Subsequently, these treated cells were transferred into a 100% embedding medium and kept at 60 °C for polymerization for 24 h. The cells embedded in the resin were finally subjected to ultrathin cutting and stained with uranium acetate for TEM sample analysis.

In addition to cells, emulsions were characterized by SEM to understand the structure of emulsion droplets. To simplify the characterization, emulsion droplets were crosslinked into particles by glutaraldehyde according to a reported protocol.[7] For this purpose, a typical emulsion (200 μL) was obtained through mixing coated cells with water and cyclopentyl methyl ether (CPME) (see the procedure described in the manuscript). Subsequently, the emulsion was crosslinked into robust particles for easy observation by SEM. The crosslinking was achieved in three steps: First, 5 μL triethylamine and 20 μL glutaraldehyde were respectively added into the emulsion for 4h crosslinking at room temperature; second, crosslinked particles

were redispersed into absolute ethanol for solvent exchange; third, the sample was dried using the critical-point dying method for the final analysis by SEM.[8]

4. Cell viability assays

The cell culture was collected and separated into two portions at the OD_{600} 0.5. One portion was used for the artificial sporulation (OLDA: 0.65 mg/mL for the coating), and the other was used as the control. The cells were firstly dispersed in PBS buffer (10 mM, pH 7.4), and then, two dyes, the green fluorescing SYTO 9 and the red fluorescent propidium iodide, were added for the incubation for 15 min, respectively. After three-time washing by PBS buffer (10 mM, pH 7.4), the cells were subjected to fluorescent microscopy.

For the growth curve, the same number of coated cells (OLDA: 0.65 mg/mL for the coating) and uncoated cells were added to LB medium containing 100 µg/mL ampicillin, respectively. Subsequently, their OD_{600} values were recorded with time, and the experiments were conducted in triplicate.

5. Protecability against external stresses

The cells were cultured (SI, section 2.2), harvested after 5 h induction, and finally coated (SI, section 6).

5.1 UV

For the treatment by UV, the same amount of coated and uncoated cells was taken into 1 mL KPi buffer (100 mM, pH 8), and subjected to UV irradiation (254 nm, 8 W) for different periods. At each time interval, 100 µL of the suspension was withdrawn and subjected to the viability assay as described in section 4 (SI).

For the enzyme activity, 100 µL of the suspension was withdrawn at each time interval. Benzaldehyde was added as the substrate to a final concentration of 100 mM. The reaction was proceeded for 10 min and extracted by adding 500 µL ethyl acetate. Then, 200 µL ethyl acetate was withdrawn and dried with anhydrous magnesium sulphate. After centrifugation, the supernatant was analyzed by GC.

5.2 Interfacial stress

For the treatment by interfacial stress, the same amount of coated and uncoated cells was taken into 1 mL KPi buffer (100 mM, pH 8). Then, 1 mL toluene was added to the cell suspensions, followed by constantly mixing by a shaker to create interfacial contact between two phases.

For the uncoated cells, 100 µL of the water phase was taken at each time interval for viability assay, and another 100 µL of the water phase was taken for enzymatic activity test as described above.

For the coated cells, 200 µL of the emulsion was taken at each time interval. The cells were collected by centrifuge and re-suspended in 100 µL KPi buffer (100 mM, pH 8) for viability assay. Another 200 µL emulsion was taken at each time interval. The cells were collected by centrifuge and re-suspended in 100 µL KPi buffer (100 mM, pH 8) for enzymatic activity test.

5.3 Acetonitrile

For the treatment by acetonitrile, same amount of coated and uncoated cells was taken and dispersed in the 0.95 mL KPi buffer (100 mM, pH 8), followed by adding 0.05 mL acetonitrile.

At each time interval, 100 µL of the suspension was withdrawn and subjected to the viability assay. And another 100 µL of the suspension was withdrawn at each time interval for the enzymatic activity test.

5.4 Heating

For the treatment by heating, same amount of coated and uncoated cells was taken and dispersed in 1 mL KPi buffer (100 mM, pH 8). The cell suspensions were then heated at 40 °C.

At each time interval, 100 µL of the suspension was withdrawn and subjected to the viability assay. And another 100 µL of the suspension was withdrawn at each time interval for the enzymatic activity test.

6. Catalytic performance

To evaluate the catalytic performance, enzyme activity (U) was defined as millimolar product formed per minute, U = $mM_{product}$ min^{-1}.

To prepare the artificial spores, *E. coli* cells were suspended in 20 mL Tris buffer (10 mM, pH 8.5) to a final optical density (OD_{600}) of 2.0, and then a mixture of DA (26 mg) and OLDA (14 mg) was dissolved in 2 mL DMSO and added to coat the cells for 2 h. Subsequently, the coated cells were collected by centrifugation and washed 3 times. The cells were finally collected and re-suspended in 5 mL PBS buffer (10 mM, pH 7.4) containing 1 mM glucose as a stock solution, which corresponds to the centration of 6.4 × 10^9 cells/mL for further use.[9]

For a fair comparison, the same amount of uncoated *E. coli* cells was required to be used in the control experiments. To this end, the uncoated *E. coli* cells were suspended in 20 mL Tris buffer (10 mM, pH 8.5) to a final optical density (OD_{600}) of 2.0, followed by centrifugation to collect the cells. The collected cells were then re-suspended in 5 mL PBS buffer (10 mM, pH 7.4) containing 1 mM glucose as a stock solution, which corresponds to the centration of 6.4 × 10^9 cells/mL for further use.

6.1. Emulsions with different oil-to-water ratios

To optimize emulsion catalysis, we prepared emulsions with different oil-to-water ratios. Typically, 0.5 mL artificial spores were first taken from the stock solution, collected by centrifugation, and then used as emulsifiers. By adding different amounts of water and organic solvents to a total volume of 0.5 mL, emulsions were obtained with gentle handshaking. These different emulsions were then applied to catalyze esterification reactions using *Candida antarctica* lipase B (CalB). The reactions were initiated upon providing a final concentration of 100 mM substrates, octanol and octanoic acid, to the emulsion mixture. At each time interval, 20 µL emulsion was withdrawn and extracted with 180 µL ethyl acetate. The ethyl acetate phase was dried with anhydrous magnesium sulfate. After centrifugation, 100 µL supernatant was subjected to gas chromatography (GC) analysis. The experiment was repeated in triplicate.

6.2. Interfacial catalysis with CalB

For interfacial catalysis with CalB, emulsions were prepared by handshaking the mixture of 0.5 mL solution of CPME and 0.5 mL solution of PBS buffer containing artificial spores with CalB. The esterification was initiated when adding 15.8 μL octanol and 15.8 μL octanoic acid. As the same as the above (i.e., 5.1), reaction products were extracted and purified from the emulsions, and finally analyzed by GC. All experiments are repeated in triplicate. Next, two control experiments were prepared for comparison. The first was the emulsions stabilized by silica particle (Sipernat®D17, donated from Evonik), where all preparation conditions were the same as the artificial spore-promoted emulsions except that uncoated cells (the same amount) were encapsulated inside as the catalysts. These emulsions were prepared according to the protocol in the literature.[10] Generally, 30 mg silica particles were mixed with 0.5 mL CPME and 0.5 mL uncoated cell solution, followed by 30-second sonification, to form the emulsions. Subsequently, 15.8 μL octanol and 15.8 μL octanoic acid were added to initiate the reaction. At each time interval, 40 μL emulsion was withdrawn and extracted with 160 μL ethyl acetate. The ethyl acetate phase was dried with anhydrous magnesium sulfate. After centrifugation, 100 μL supernatant was subjected to GC analysis. The experiment was repeated in triplicate. For the second control, a two-phase system was prepared by only mixing 0.5 mL CMPE phase with 0.5 mL PBS buffer containing the same amount of uncoated cells. Then the two-phase was evaluated for the same esterification reaction as described above using GC analysis. All samples are were prepared in triplicate.

6.3. Interfacial catalysis with different concentrations of artificial spores

To further evaluate interfacial catalysis, Pickering emulsions were stabilized by the CalB-containing artificial spores (0.65 mg/mL OLDA) with the concentration of 3.2×10^9 cells/mL, 6.4×10^9 cells/mL, 12.8×10^9 cells/mL and 16.0×10^9 cells/mL, respectively. Subsequently, 15.8 μL octanol and 15.8 μL octanoic acid were added to initiate the reaction. At each time interval, 20 μL emulsion was withdrawn and extracted with 380 μL ethyl acetate. The ethyl acetate phase was dried with anhydrous magnesium sulfate. After centrifugation, 100 μL

supernatant was subjected to gas chromatography (GC) analysis. The experiment was repeated in triplicate.

6.4. Interfacial catalysis with different hydrophobicity of artificial spores

For the cells containing CalB, different concentrations of OLDA (0.4, 0.65, 1.05, and 1.4 mg/mL) were used to form artificial spores with different hydrophobicity. After coating, the cells were washed by water 3 times, collected by centrifuge and dispersed in 0.5 mL PBS buffer. Afterwards, 0.5 mL CPME was added for emulsions, respectively. Subsequently, 15.8 μL octanol and 15.8 μL octanoic acid were added to initiate the reaction. At each time interval, 20 μL emulsion was withdrawn and extracted with 380 μL ethyl acetate. The ethyl acetate phase was dried with anhydrous magnesium sulfate. After centrifugation, 100 μL supernatant was subjected to gas chromatography (GC) analysis. The experiment was repeated in triplicate.

6.5. Interfacial catalysis with BAL

For the interfacial catalysis with BAL, emulsions were prepared by handshaking the mixture of 0.5 mL solution of CPME and 0.5 mL PBS buffer of BAL-containing artificial spores. The benzoin condensation reaction was initiated when adding 10.1 μL benzaldehyde. At each time interval, 20 μL emulsion was withdrawn and then extracted with 180 μL ethyl acetate for product extraction. The ethyl acetate phase was dried with anhydrous magnesium sulfate. After centrifugation, 100 μL supernatant was subjected to GC analysis. The experiment was repeated in triplicate. For the control, the two-phase system was prepared by only mixing 0.5 mL CMPE phase with 0.5 mL PBS buffer containing the same amount of uncoated cells. Afterwards, 10.1 μL benzyl aldehyde was added to initiate the reaction. At each time interval, 10 μL organic phase was withdrawn and then added to 90 μL ethyl acetate for product extraction. The ethyl acetate phase was dried with anhydrous magnesium sulfate. After centrifugation, about 100 μL supernatant was subjected to GC analysis. The experiment was repeated in triplicate.

6.6. Interfacial catalysis with ADH-a

For interfacial catalysis with ADH-a, a cofactor-dependent reaction was performed. Typically, 0.5 mL artificial spore PBS buffer solution and 0.5 mL CPME were shaken by hand for the formation of emulsions. After emulsification, 11.7 μL acetophenone solution was added to initiate the reduction reaction without the supplement of the cofactor. At each time interval, 20 μL emulsion was withdrawn and extracted with 180 μL ethyl acetate. The ethyl acetate phase was dried with anhydrous magnesium sulfate. After centrifugation, about 100 μL supernatant was subjected to GC analysis. The experiment was repeated in triplicate. For the two-phase control, 0.5 mL uncoated cells PBS buffer solution were mixed with 0.5 mL CPME. Afterwards, 11.7 μL acetophenone was added to initiate the reaction without adding cofactor. At each time interval, 10 μL organic phase was withdrawn and mixed with 90 μL ethyl acetate for product extraction. The ethyl acetate phase was then dried with anhydrous magnesium sulfate. After centrifugation, 100 μL supernatant was taken for GC measurement. The experiment was repeated in triplicate.

6.7. Multienzyme cascade with ADH-ht and CalB

For the cascade reaction with ADH-ht and CalB (see Figure 6a in manuscript), the procedure is similar to the above. Typically, 0.5 mL CPME was added to the aqueous mixture of 0.25 mL PBS buffer solution of artificial spores containing ADH-ht and 0.25 mL PBS buffer solution of artificial spores containing CalB. After the same way as the above to make the mixture into emulsions, two substrates, 15.8 μL octanal and 15.8 μL octanoic acid, were simultaneously added to initiate the multienzyme cascade reaction without using cofactor. At each time interval, the reaction products were purified in the same way as the single-step reaction described above. For the two-phase control, 0.5 mL CPME was added to 0.25 mL PBS buffer solution of uncoated cells containing ADH-ht and 0.25 mL PBS buffer solution of uncoated cells containing CalB. Afterwards, 15.8 μL octanal and15.8 μL octanoic acid were added to initiate the reaction also without using cofactor. At each time interval, the organic phase (10 μL) was withdrawn and added to 90 μL ethyl acetate for product extraction. The ethyl acetate phase was dried

with anhydrous magnesium sulfate. After centrifugation, 100 μL supernatant was subjected to GC analysis. The experiment was repeated in triplicate.

6.8. Multienzyme cascade with ADH-ht and BAL

To prepare the cascade reactions with ADH-ht and BAL, all the procedures are the same as the cascade with ADH-ht and CalB except that artificial spores containing BAL were used to replace artificial spores containing CalB. To initiate the reaction, 5.4 μL ethanol and 10.1 μL benzaldehyde were added to 1 mL emulsions without cofactors (see Figure 6b in manuscript). Their products were obtained and analyzed in the way as the above. Again, in the similar way, a two-phase system was prepared without cofactor, and reactions were performed and analyzed accordingly.

6.9. Multienzyme cascade with CalB and BAL

For the cascade reaction with CalB and BAL, the emulsions were prepared in the same way as the protocol in 5.5 and 5.6, where the only difference was coated cells containing CalB and BAL in use. For their cascade reaction, 17 μL vinyl n-hexanoate,15.8 μL octanoic acid and 10.1 μL benzyl aldehyde were added to initiate the reaction. The two-phase control was prepared in the same way as the protocol described in 5.5 and 5.6. The experiment was repeated in triplicate.

6.10. Chemoenzymatic cascade with palladium nanoparticles and CalB

For the chemoenzymatic cascade reaction with CalB and palladium nanoparticles (Pd NPs), 0.5 mL CPME was added to 0.5 mL PBS buffer solution of artificial spores containing CalB and Pd NPs (see Figure 7 in manuscript). After being shaken by hand for emulsification, 17 μL vinyl n-hexanoate and 10.1 μL benzyl aldehyde were added to initiate the reaction under a hydrogen atmosphere. At each time interval, 20 μL emulsion was withdrawn and then extracted with 180 μL ethyl acetate. The ethyl acetate phase was dried with anhydrous magnesium sulfate. After centrifugation, 100 μL supernatant was subjected to GC analysis. The experiment was repeated in triplicate. For the two-phase control, 0.5 mL CPME was added to 0.5 mL PBS buffer of artificial spores containing CalB and Pd NPs without emulsification. Afterwards, 17 μL

vinyl n-hexanoate and 10.1 μL benzyl aldehyde were added to initiate the reaction under a hydrogen atmosphere. At each time interval, 10 μL organic phase was withdrawn and added to 90 μL ethyl acetate for product extraction. The ethyl acetate phase was dried with anhydrous magnesium sulfate. After centrifugation, 100 μL supernatant was subjected to GC analysis. The experiment was repeated in triplicate.

7. Experimental data

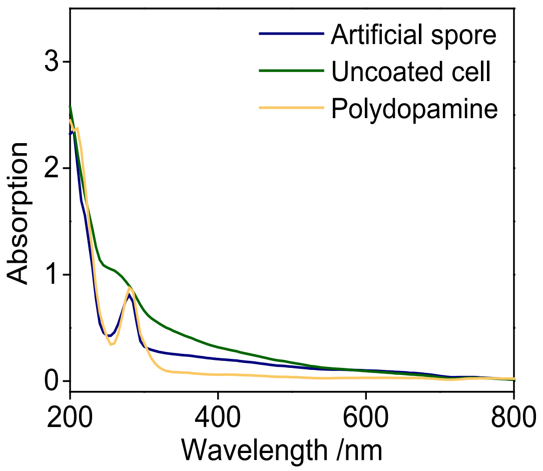

Supplementary Figure 1. UV-VIS spectra of the polydopamine (OLDA: 0.65 mg/mL) alone, uncoated *E. coli* cells and artificial spores (OLDA: 0.65 mg/mL).

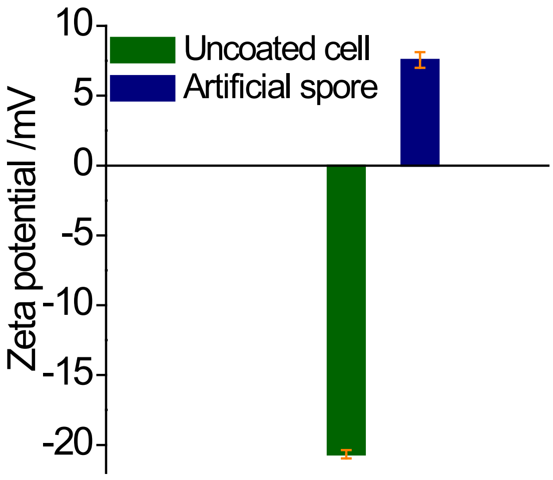

Supplementary Figure 2. Zeta potential of the uncoated and coated (OLDA: 0.65 mg/mL) *E. coli* cells. The results are the average values of three parallel experiments. The error bars represent the standard deviations of three parallel measurements, n=3.

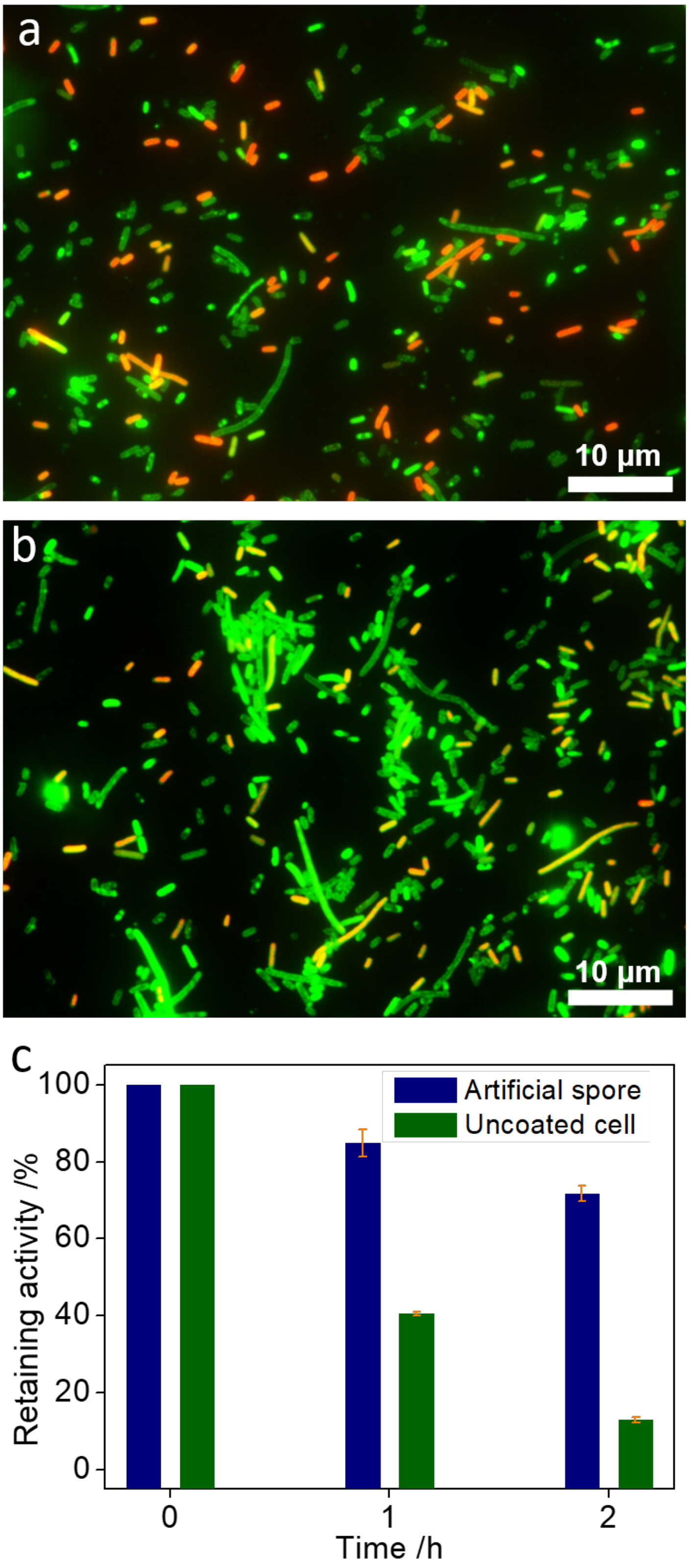

Supplementary Figure 3. Live/dead assay of heat-treated (40 °C, 2 h) cells: a) uncoated *E. coli* cells, b) artificial spores, green - live cells; red - dead cells. c) BAL activity of heat-treated (40 °C) cells. The results in (c) are the average values of three parallel experiments. The error bars represent the standard deviations of three parallel measurements, n=3.

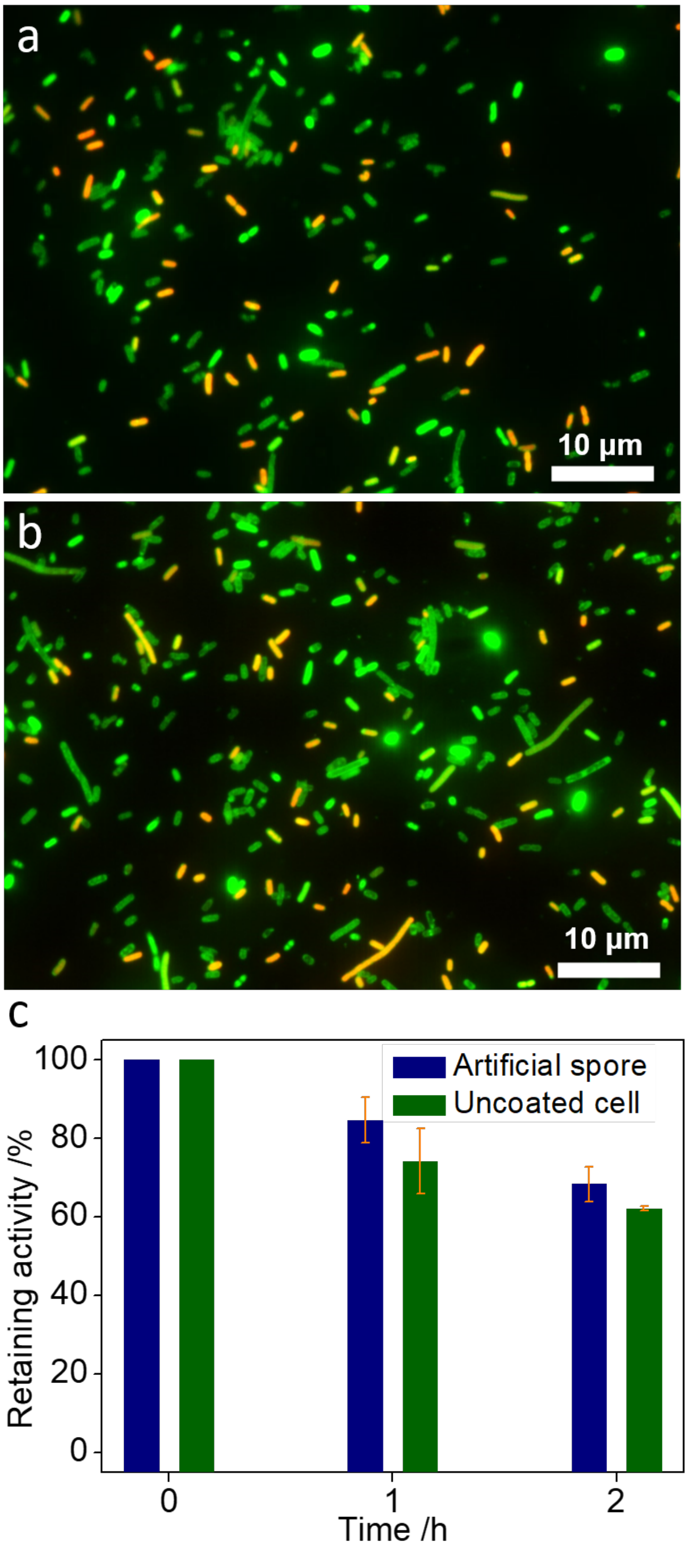

Supplementary Figure 4. Live/dead assay of 5% acetonitrile-treated cells (2 h): a) uncoated *E. coli* cells, b) artificial spores, green - live cells; red - dead cells. c) BAL activity of 5% acetonitrile-treated cells. The results in (c) are the average values of three parallel experiments. The error bars represent the standard deviations of three parallel measurements, n=3.

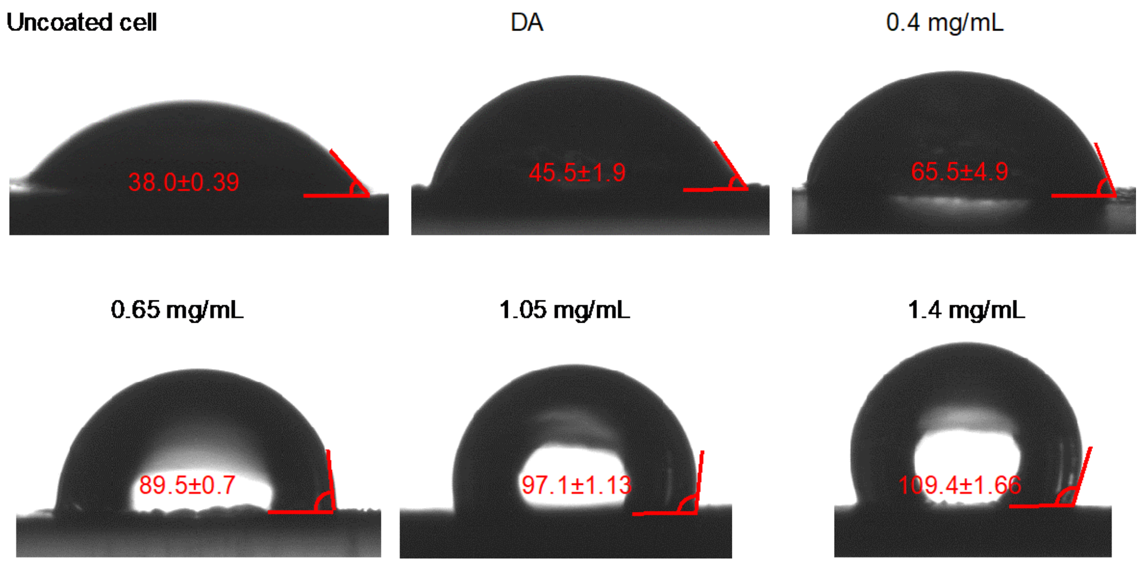

Supplementary Figure 5. Contact angle of uncoated *E. coli* cells and artificial spores with different OLDA concentrations, total coating concentrations are 2 mg/mL. The results are the average values of three parallel experiments. The error bars (±) represent the standard deviations of three parallel measurements, n=3.

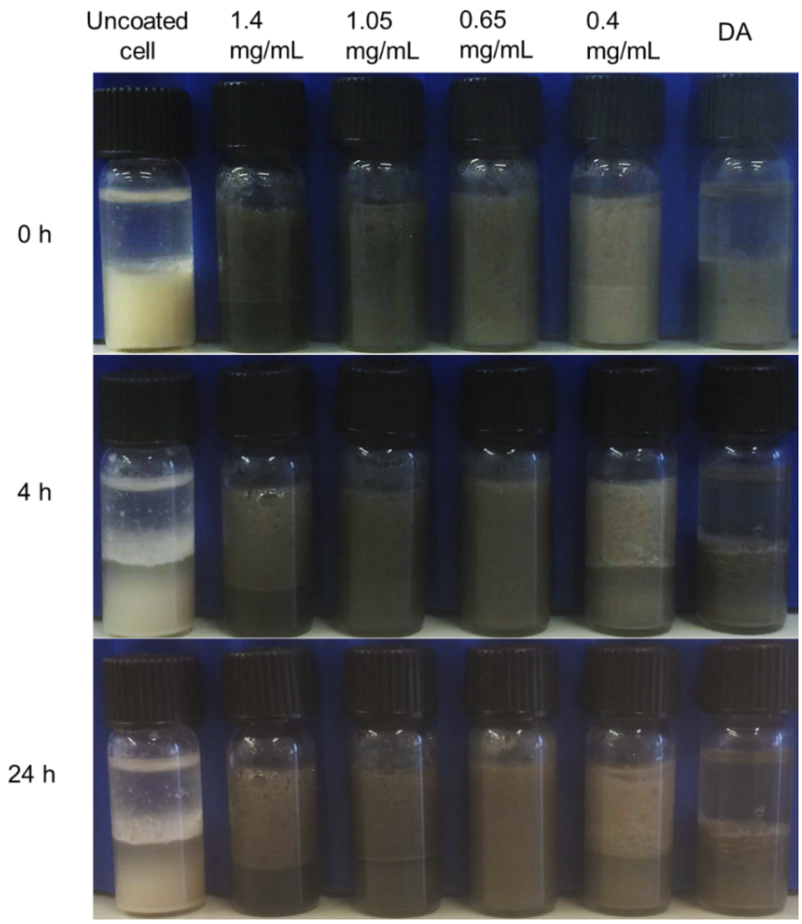

Supplementary Figure 6. Emulsion appearance and stability from uncoated *E. coli* cells and artificial spores with different OLDA concentrations, total coating concentrations are 2 mg/mL.

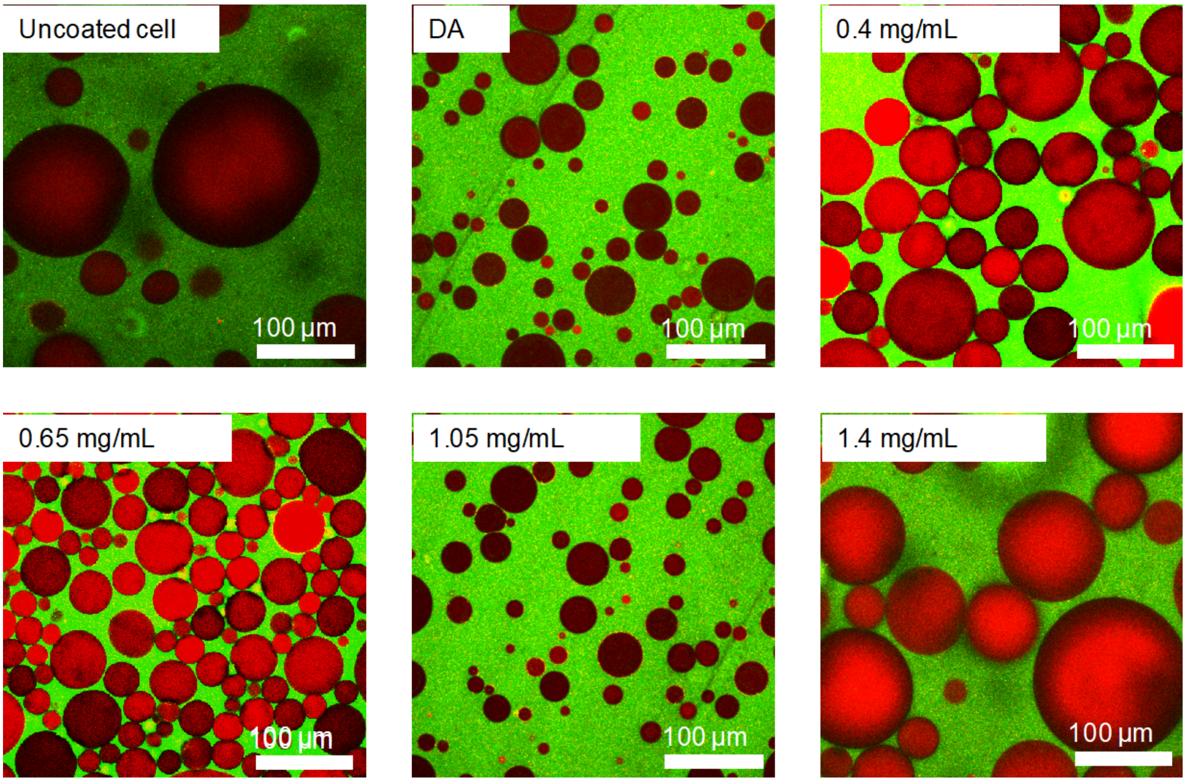

Supplementary Figure 7. Emulsion droplets from uncoated *E. coli* cells and artificial spores with different OLDA concentrations, total coating concentrations are 2 mg/mL; red – organic phase, green – aqueous phase.

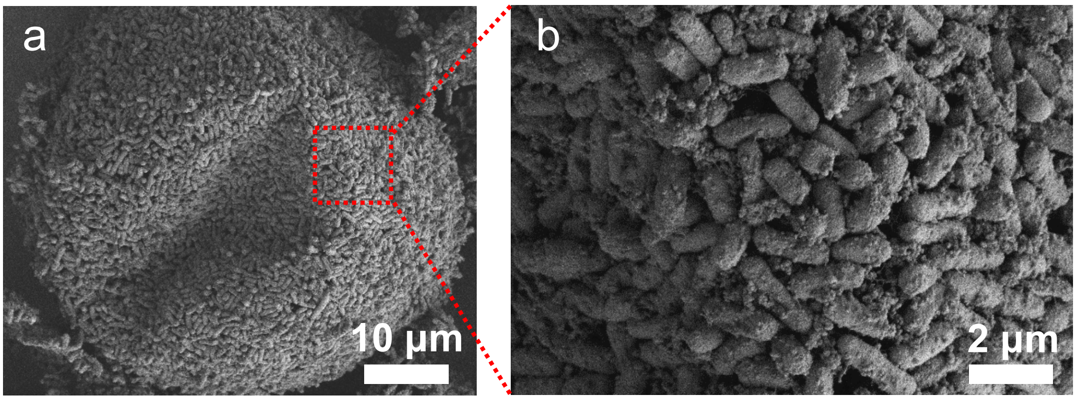

Supplementary Figure 8. SEM images of emulsion droplets stabilized by artificial spores (OLDA: 0.65 mg/mL) at different magnifications.

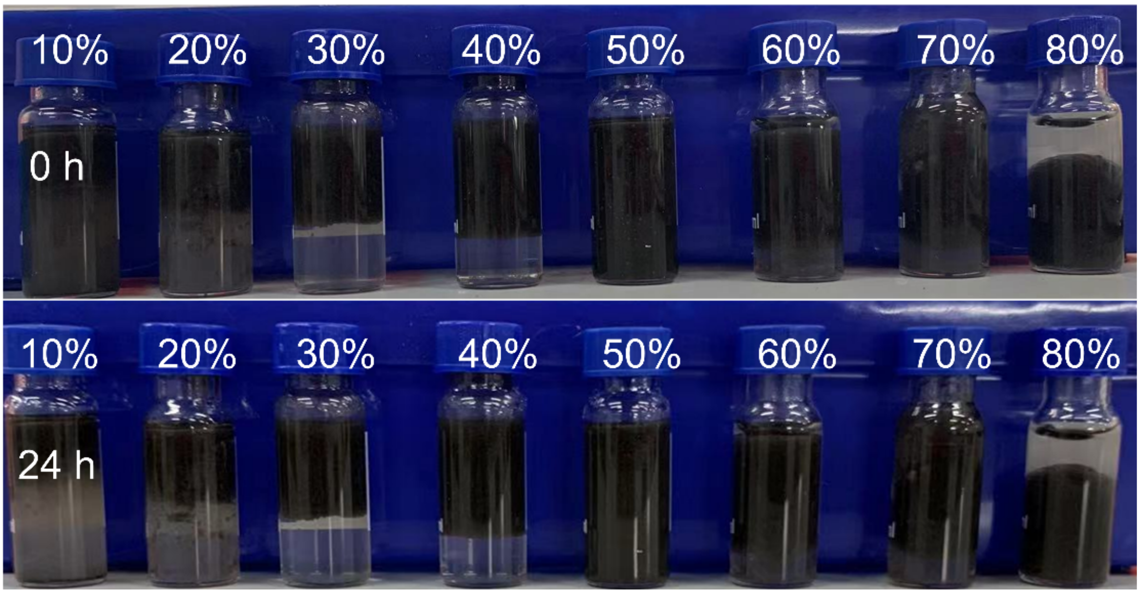

Supplementary Figure 9. The stability of emulsions stabilized by artificial spores (OLDA: 0.65 mg/mL) with different organic fractions from 10% to 80%.

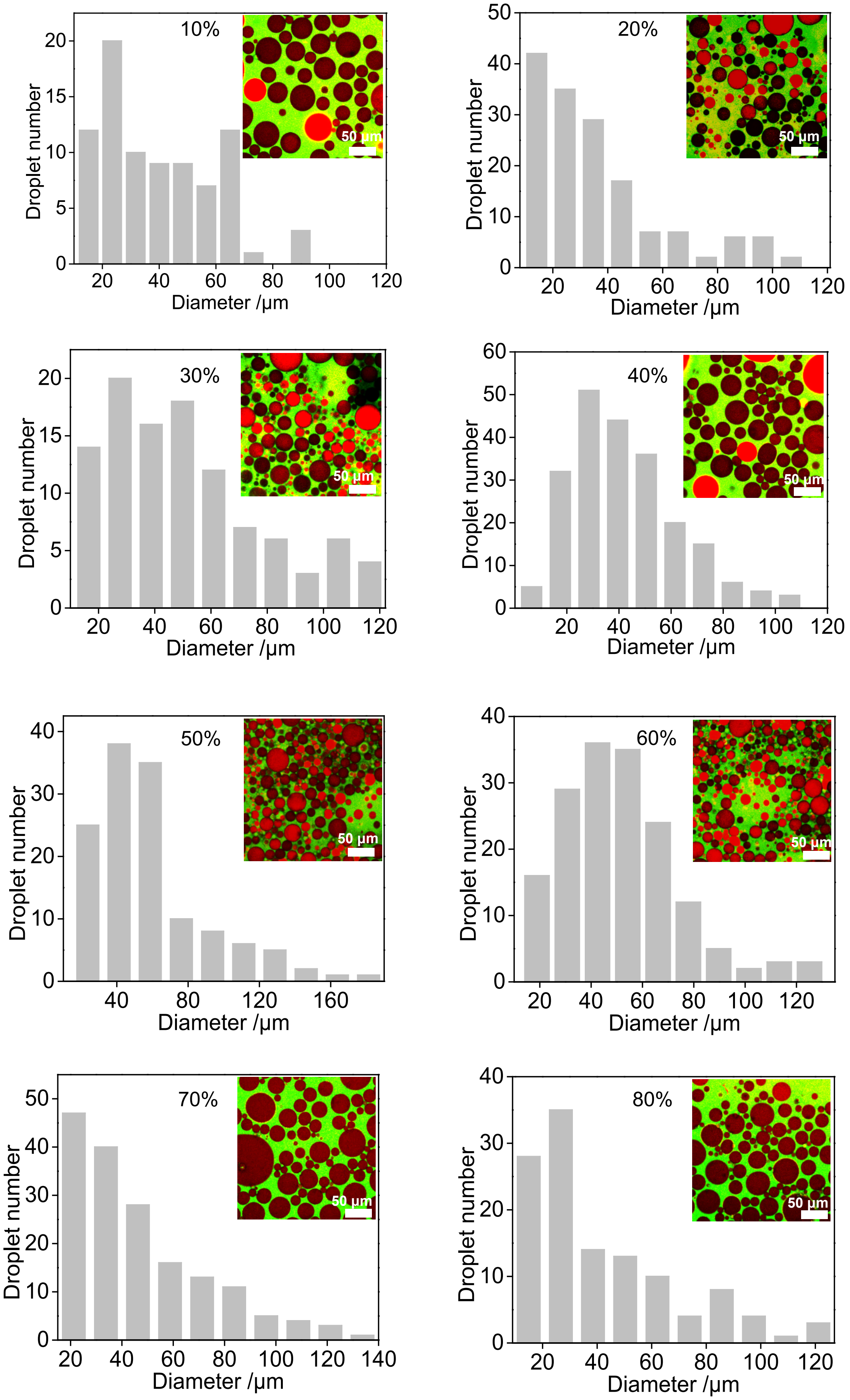
10%
20%
30%
40%
50%
60%
70%
80%
50 μm
Droplet number
Diameter /μm

Supplementary Figure 10. The number and size distribution of emulsion droplets stabilized by artificial spores (OLDA: 0.65 mg/mL) with different organic fractions from 10% to 80%. Inserts are the CLSM images of these emulsions; red – organic phase, green – aqueous phase.

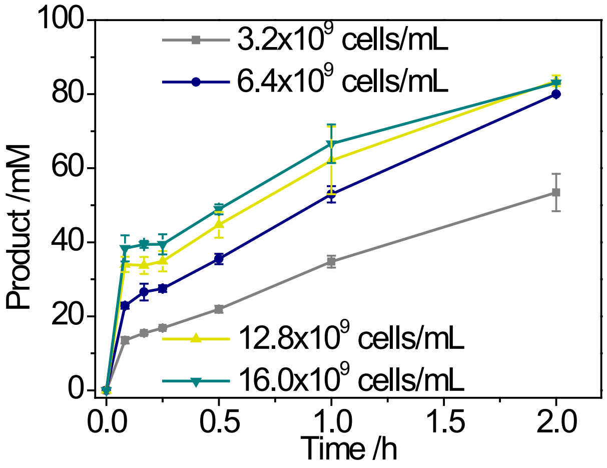

Supplementary Figure 11. Reaction profiles of artificial spores (OLDA: 0.65 mg/mL) with different concentrations. The results are the average values of three parallel experiments. The error bars represent the standard deviations of three parallel measurements, n=3.

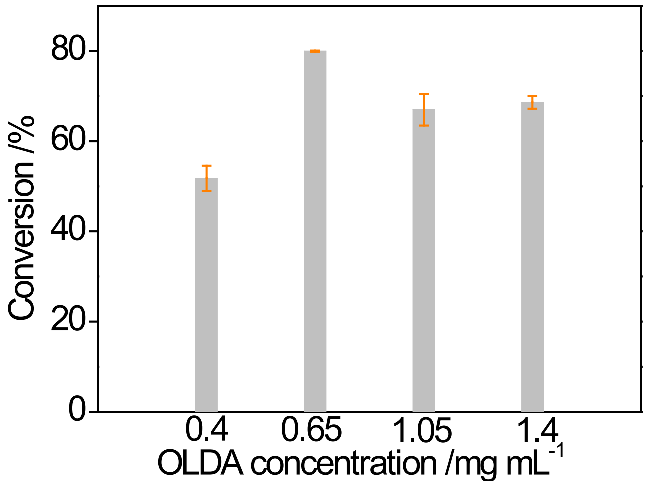

Supplementary Figure 12. Conversion of artificial spores with different hydrophobicity after 2 h reaction. The results are the average values of three parallel experiments. The error bars represent the standard deviations of three parallel measurements, n=3.

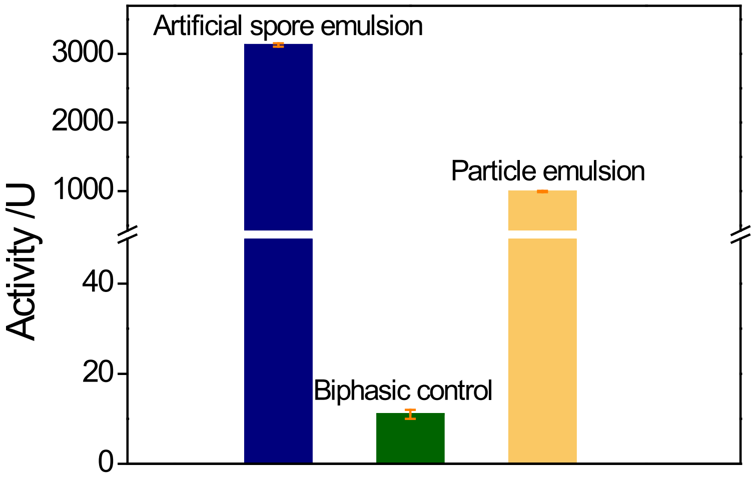

Supplementary Figure 13. Activity comparison of the reaction catalyzed by CalB in different systems. Blue column: artificial spores-stabilized emulsion; green column: biphasic control with uncoated *E. coli* cells; yellow column: particles-stabilized emulsion with uncoated *E. coli* cells. The results are the average values of three parallel experiments. The error bars represent the standard deviations of three parallel measurements, n=3.

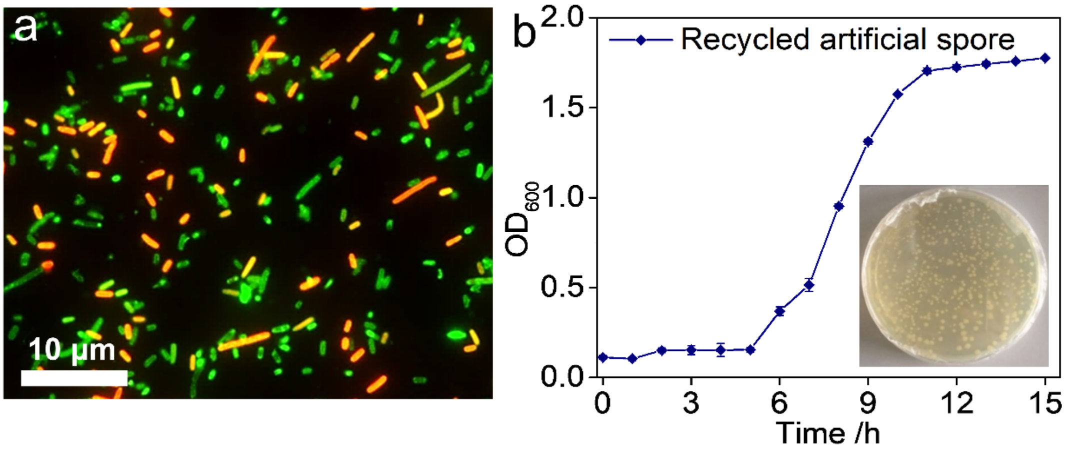

Supplementary Figure 14. a) Live/dead assay of the second-time recycled artificial spores; green - live cells; red - dead cells. b) Growth curve of the second-time recycled artificial spores and their growth on agar plate. The results in (b) are the average values of three parallel experiments. The error bars represent the standard deviations of three parallel measurements, n=3.

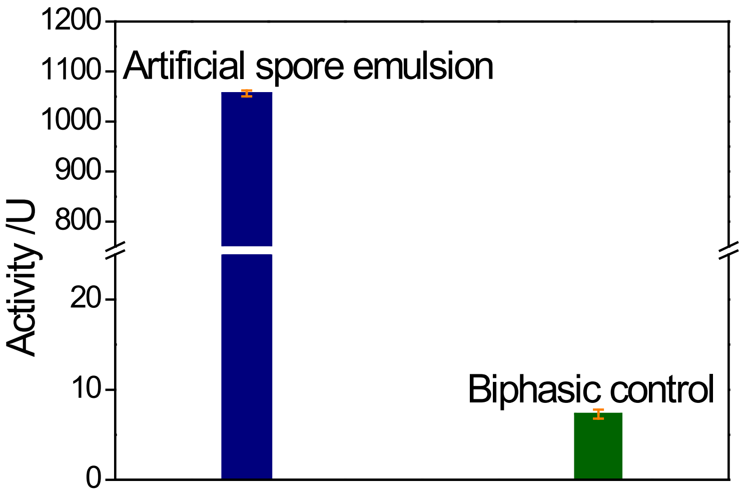

Supplementary Figure 15. Activity comparison of model reaction catalyzed by BAL in different systems. Blue column: artificial spores-stabilized emulsion; green column: biphasic control with uncoated *E. coli* cells. The results are the average values of three parallel experiments. The error bars represent the standard deviations of three parallel measurements, n=3.

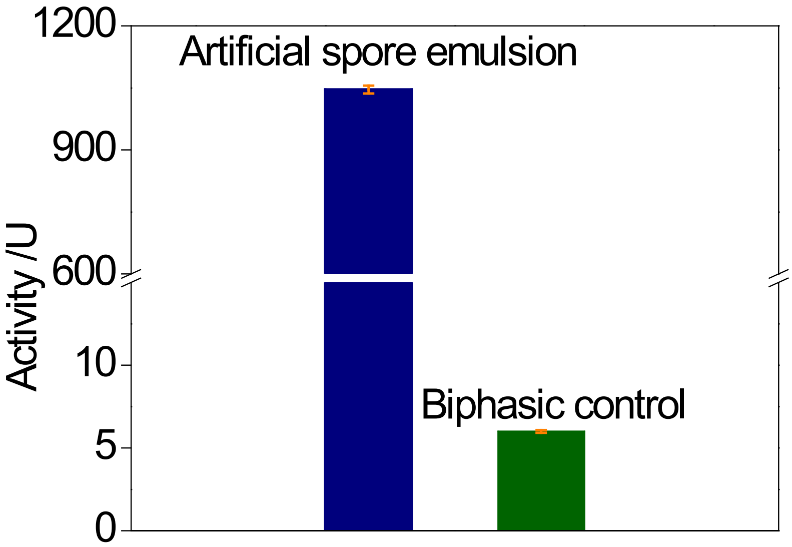

Supplementary Figure 16. Activity comparison of model reaction catalyzed by ADH-a in different systems. Blue column: artificial spores-stabilized emulsion; green column: biphasic

control with uncoated *E. coli* cells. The results are the average values of three parallel experiments. The error bars represent the standard deviations of three parallel measurements, n=3.

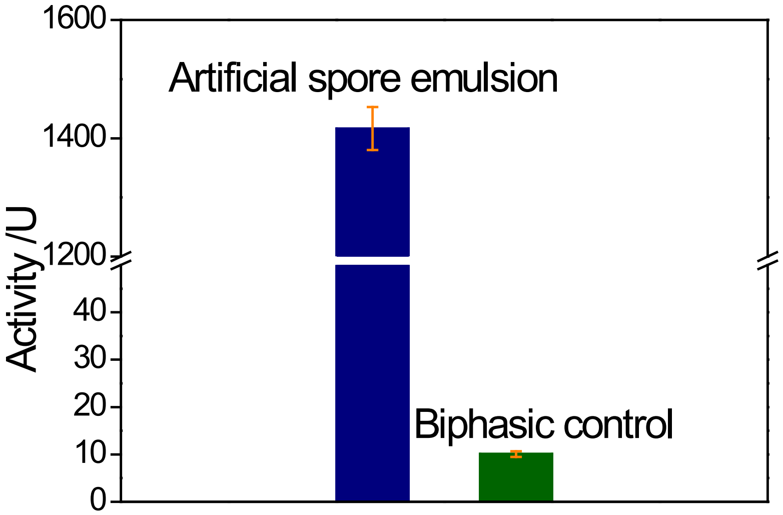

Supplementary Figure 17. Activity comparison of ADH-ht and CalB cascades in different systems. Blue column: artificial spores-stabilized emulsion; green column: biphasic control with uncoated *E. coli* cells. The results are the average values of three parallel experiments. The error bars represent the standard deviations of three parallel measurements, n=3.

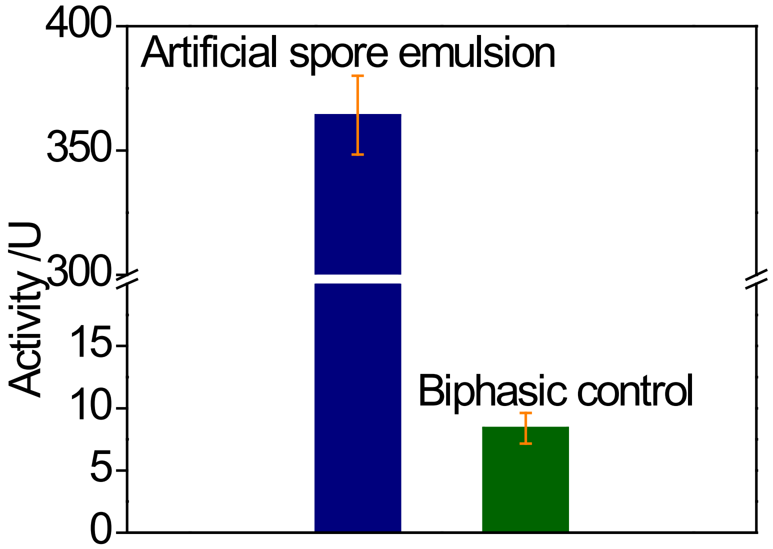

Supplementary Figure 18. Activity comparison of ADH-ht and BAL cascades in different systems. Blue column: artificial spores-stabilized emulsion; green column: biphasic control with uncoated *E. coli* cells. The results are the average values of three parallel experiments. The error bars represent the standard deviations of three parallel measurements.

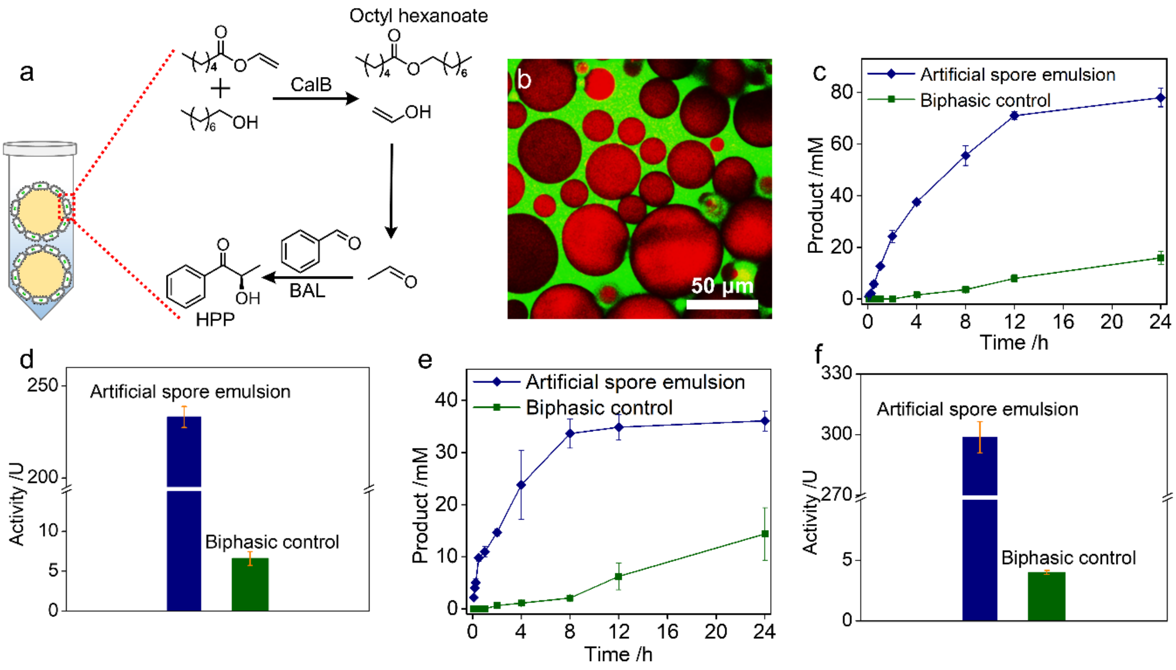

Supplementary Figure 19. a) Reaction scheme of cascade reaction catalyzed by CalB and BAL. b) CLSM image of the emulsion stabilized by artificial spores containing CalB and BAL; red – organic phase, green – aqueous phase. c) The reaction profiles of transesterification reaction by CalB, based on the production of octyl hexanoate. d) the activity comparison in different systems, based on the production of octyl hexanoate. e) The reaction profiles of cascade reaction by CalB and BAL, based on the production of (*R*)-2-hydroxypropiophenone (HPP). f) The activity comparison in different systems, based on the production of (*R*)-2-hydroxypropiophenone. The results in (c, d, e, f) are the average values of three parallel experiments. The error bars represent the standard deviations of three parallel measurements, n=3.

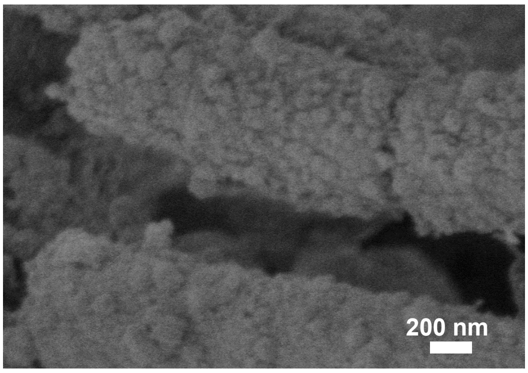

Supplementary Figure 20. SEM image of Pd@AS.

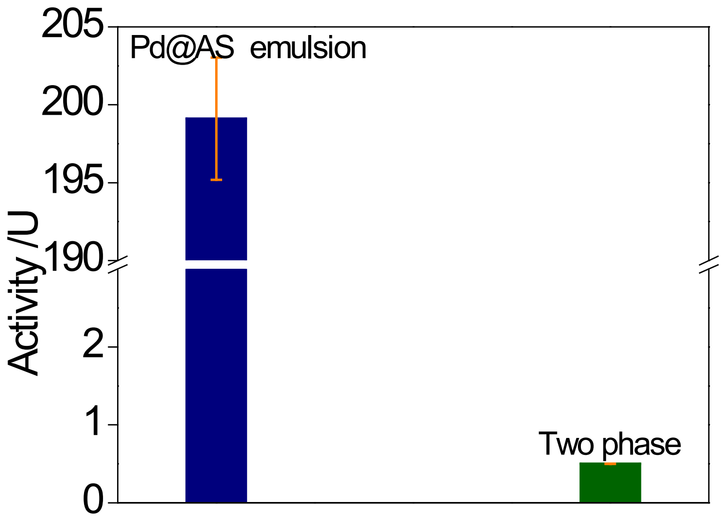

Supplementary Figure 21. Activity comparison of Pd and CalB cascade reaction in different systems. The results are the average values of three parallel experiments. The error bars represent the standard deviations of three parallel measurements, n=3.

8. References

1 Alghunaim, A., Kirdponpattara, S. & Newby, B.m. Z. Techniques for determining contact angle and wettability of powders. *Powder Technol.* **287**, 201-215 (2016).

2 Studier, F. W. Protein production by auto-induction in high-density shaking cultures. *Protein Expr. Purif.* **41**, 207-234 (2005).

3 Sun, Z., Glebe, U., Charan, H., Boker, A. & Wu, C. Enzyme-polymer conjugates as robust Pickering interfacial biocatalysts for efficient biotransformations and one-pot cascade reactions. *Angew. Chem. In.t Ed.* **57**, 13810-13814 (2018).

4 de Gonzalo, G., Lavandera, I., Faber, K. & Kroutil, W. Enzymatic reduction of ketones in "micro-aqueous" media catalyzed by ADH-A from *Rhodococcus ruber*. *Org. Lett.* **9**, 2163-2166 (2007).

5 Sattler, J. H. *et al.* Introducing an in situ capping strategy in systems biocatalysis to access 6-aminohexanoic acid. *Angew. Chem. Int. Ed.* **53**, 14153-14157 (2014).

6 Cagle, G. D. Critical-point drying: rapid method for the determination of bacterial extracellular polymer and surface structures. *Appl. Microbiol.* **28**, 312-316 (1974).

7 Abay, A. *et al.* Glutaraldehyde – A subtle tool in the investigation of healthy and pathologic red blood cells. *Front. Physiol.* **10**, 514 (2019).

8 Bray, D. in *Supercritical fluid methods and protocols* (eds John R. Williams & Anthony A. Clifford) 235-243 (Humana Press, 2000).

9 Volkmer, B. & Heinemann, M. Condition-dependent cell volume and concentration of *Escherichia coli* to facilitate data conversion for systems biology modeling. *PLoS One* **6**, e23126 (2011).

10 Zhao, Q., Ansorge-Schumacher, M. B., Haag, R. & Wu, C. Living whole-cell catalysis in compartmentalized emulsion. *Bioresour. Technol.* **295**, 122221 (2020).
